# Insight into the prospects and limitations of mechanochemically-synthesised lithium tetrahalogallates, LiGaX_4_ (X = Cl, Br, I), as Li-ion conductors

**DOI:** 10.1039/d5sc03999a

**Published:** 2025-11-06

**Authors:** Nicolás Flores-González, Martí López, Nicolò Minafra, Jamie Jack, Jan Bohnenberger, Atsushi Inoishi, Nalin Gupta, Leandro Liborio, Francesc Viñes, Ronald I. Smith, Peter J. Baker, Ingo Krossing, Wolfgang G. Zeier, Francesc Illas, Duncan H. Gregory

**Affiliations:** a WestCHEM, School of Chemistry, University of Glasgow Joseph Black Building Glasgow G12 8QQ UK Duncan.Gregory@glasgow.ac.uk; b Departament de Ciència de Materials i Química Física & Institut de Química Teòrica i Computacional (IQTCUB), Universitat de Barcelona c/Martí i Franquès 1-11 08028 Barcelona Spain; c Institute for Inorganic and Analytical Chemistry, University of Münster Correnstr. 39 48149 Germany; d Institute of Energy Materials and Devices (IMD), Helmholtz-Institut Münster (IMD-4: HI MS), Forschungszentrum Jülich Münster D-48149 Germany; e Institut für Anorganische und Analytische Chemie and Freiburger Materialforschungszentrum (FMF), Universität Freiburg Albertstr. 21 79104 Freiburg Germany; f Institute for Materials Chemistry and Engineering, Kyushu University Kasuga-koen 6-1, Kasuga Fukuoka 816-8580 Japan; g ISIS Pulsed Neutron and Muon Source, STFC Rutherford Appleton Laboratory Didcot Oxfordshire OX11 0QX UK; h Department of Materials Engineering, Faculty of Engineering, University of Concepción Concepción 4070415 Chile

## Abstract

Halide solid-state electrolytes have attracted significant interest due to their appreciable Li^+^ conductivity at room temperature, good electrochemical stability against oxidation, and favourable compatibility with oxide cathodes. Nevertheless, the family of lithium tetrahalogallates, LiGaX_4_ (X = Cl, Br, I), has scarcely been studied and, consequently, their physicochemical properties remained largely unknown. In this work, we report the mechanochemical synthesis of high-purity LiGaX_4_ and investigate their crystal structures, thermal, electronic, vibrational, and ionic transport properties through a combination of advanced characterisation techniques and computational methods. Powder X-ray and neutron diffraction confirm that all three phases crystallise in a monoclinic unit cell (*P*2_1_/*c*), isostructural to LiAlX_4_ analogues. Preliminary results indicate that LiGaBr_4_ exhibits the highest ionic conductivity at room temperature (4.87 × 10^−6^ S cm^−1^) among the series. Compared to LiAlX_4_, the diffusion pathways in LiGaX_4_ showed a lower dimensionality and higher activation energies for Li^+^ diffusion, which results in reduced ionic conductivities. Periodic density functional theory (DFT) based calculations indicate a general correlation between computed band gaps and electrochemical windows in LiMX_4_ materials (M = Al, Ga; X = Cl, Br, I). Additionally, μ^+^SR data demonstrate that softer lattices provide lower activation energies for Li^+^ migration and suggest that additional factors influence the results obtained through electrochemical impedance spectroscopy.

## Abstract 2

Advanced characterisation of mechanochemically synthesised LiGaX_4_ halides uncovers their structures, transport properties and key differences between short- and long-range conduction.

## Graphical abstract

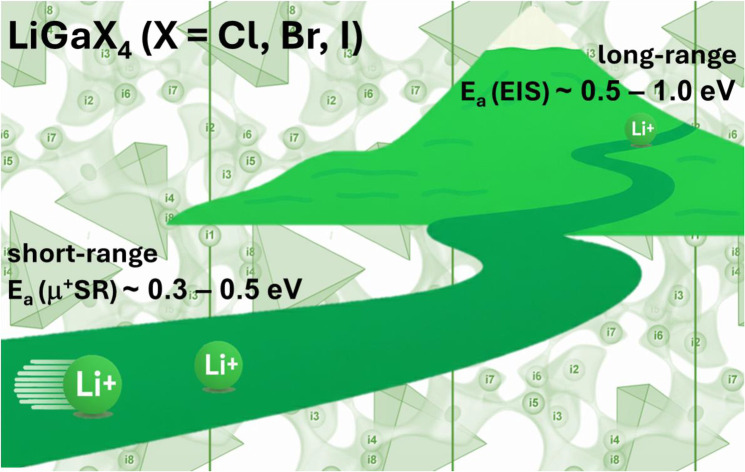

## Introduction

Halide solid-state electrolytes (HSEs)^1,2^ of general composition Li_*a*_MX_*b*_ (M = metal, X = F, Cl, Br, I), such as LiAlX_4_ (X = halogen),^3^ Li_*x*_ScCl_3+*x*_,^4^ Li_2_M_2/3_Cl_4_ (M = Sc, Cr),^5,6^ Li_2_In_*x*_Sc_2/3−*x*_Cl_4_,^7^ Li_2_ZrCl_6_,^8^ Li_3_MX_6_ (M = Sc, Y, In, La, Ho, Er; X = halogen),^9–15^ Li_3−*x*_M^III^_1−*x*_M^IV^_*x*_Cl_6_ (M^III^ = Y, Ho, Er, Yb, Lu; M^IV^ = Zr, Hf),^16–19^ as well as argyrodite chalcogenide halides, Li_7−*y*_PS_6−*y*_X_*y*_ (*y* = 0–2, X = Cl, Br),^20^ and oxihalides, LiMOCl_4_ (M = Nb, Ta),^21^ have attracted increasing attention due to their appreciable Li^+^ conductivity at room temperature, good electrochemical stability against oxidation, and favourable compatibility with oxide cathodes.

Despite the progress made with HSEs, the family of compounds LiGaX_4_ (X = Cl, Br, I) has scarcely been studied in terms of ionic conductivity. Synthesis involves heating the appropriate anhydrous salts, LiX and GaX_3_ (*e.g.* LiGaBr_4_ can be prepared by mixing LiBr and GaBr_3_ at 250 °C in a sealed glass ampoule for 2 days).^22^ Honle *et al.*^23^ reported the synthesis and crystal structures of LiGaCl_4_ and LiGaI_4_, which were shown to be isostructural to LiAlCl_4_ (*P*2_1_/*c*). Okuda *et al.*^24^ reported the ionic conductivity and structure of halocomplex salts of group 13 elements. Among them, LiGaBr_4_ prepared by conventional solid-state synthesis exhibited an ionic conductivity of 7 × 10^−6^ S cm^−1^ at 24 °C, which increases to 2.0 × 10^−5^ S cm^−1^ when synthesised by mechanochemical methods.^25^ Recently, Kahle *et al.*^26^ identified LiGaI_4_ as a potential fast-ionic conductor by high-throughput computational screening followed by full first-principles molecular dynamics calculations. However, there are no conductivity values reported for LiGaI_4_ (or for LiGaCl_4_) in the literature at the time of writing.

Motivated by the promising properties of halide salts and the lack of data on Ga-containing materials within this family, we systematically investigated the crystal structures, and thermal, electronic, vibrational, and ionic transport properties of lithium tetrahalogallates, LiGaX_4_ (X = Cl, Br, I). While their ionic conductivities were found to be significantly lower than those of more established HSEs, our study sought to rationalise how changes in M (Al^3+^*vs.* Ga^3+^) and the resulting polyanion chemistry mediate transport and stability. Such understanding is critical for the rational design of new materials and for extending the known boundaries in halide electrolytes.

In this work, we show that the tetrahalogallates can be easily synthesised in one step using mechanochemistry. We used both electrochemical impedance (EIS) and muon-spin (μ^+^SR) spectroscopies to study the Li^+^ diffusion behaviour and compare the results obtained from the two techniques. Significantly, the μ^+^SR studies have provided insight into the Li^+^ dynamics at the local level without the need to consider the microscopic and macroscopic factors typically encountered in conventional EIS analyses.

## Experimental

### Synthesis of LiGaX_4_

LiCl (Sigma-Aldrich, ≥99.99%), GaCl_3_ (Sigma-Aldrich, ultra-dry, 99.999%), LiBr (Alfa Aesar, anhydrous, 99.995%), LiI (Sigma-Aldrich, anhydrous, 99.99%) and GaI_3_ (Alfa Aesar, ultra-dry, 99.999%) were used as starting materials without further purification. GaBr_3_ (Alfa Aesar, anhydrous, ≥99%) required purification by sublimation at 80 °C under vacuum for *ca.* 2 hours before use, due to the presence of impurities (presumed to be gallium metal) as determined from the DTA trace (Fig. S1). Alternatively, GaBr_3_ (Alfa Aesar, ultra-dry, 99.998%) was used as a starting material without further purification. Because of the air and/or moisture sensitivity of the starting materials and the final products, all manipulations were carried out in a recirculating Ar-filled (BOC, 99.998%) glove box (MBraun LABstar, O_2_ < 0.5 ppm, H_2_O < 0.5 ppm). *Ca*. 0.5 g in total of the starting materials were weighed out accurately in the desired molar ratio (1 : 1) and then transferred to a stainless-steel grinding jar that was filled with ten stainless steel balls (10 mm diameter), each of which weighed *ca*. 4 g. The grinding jar was sealed under Ar before removal from the glove box. Ball milling was conducted with a ball-to-powder ratio (BPR) of 80 : 1 in a planetary ball mill (Retsch PM100) in 5-minute milling periods (reverse rotation), which were followed by 5 minute rest periods. Ball mill parameters are summarised in Table S1 and the colours of the as-synthesised products are shown in Table S2. LiGaX_4_ were found to be highly hygroscopic/deliquescent and corrosive.

## Characterisation

### Powder X-ray diffraction

Powder X-ray diffraction (PXD) data were collected at room temperature with a PANalytical Empyrean diffractometer with Cu-Kα radiation in transmission geometry. The moisture/air-sensitive samples were loaded inside glass capillaries (0.5 or 0.7 mm internal diameter) and sealed with wax in an Ar-filled recirculating glovebox. The sample capillaries were flame-sealed outside the glove box. The aligned capillaries were continuously rotated throughout the analysis and scanned over 5 ≤ 2*θ*/° ≤ 85 ranges (0.013 step size, 1 h).

### Powder neutron diffraction

Powder neutron diffraction (PND) data were collected on the Polaris powder diffractometer at the ISIS pulsed neutron and muon source, Rutherford Appleton Laboratory, UK.^27^ In order to obtain sufficient amount of material for PND measurements, samples were synthesised 3 times (*i.e*. in 3 batches) and subsequently mixed. Samples of *ca*. 1 g were loaded into a 6 mm diameter thin-walled vanadium sample can, which was sealed using an indium wire gasket, inside a glove box. The sample cans were mounted in the diffractometer and data collected at room temperature with 350 μAh integrated proton beam current to the ISIS neutron target (corresponding to ∼2 h total exposure). Data reduction, which included an empirical absorption correction, and generation of files suitable for profile refinement, used the Mantid open source software.^28^

### Rietveld analysis

Structure refinement was carried out by the Rietveld method,^29^ employing GSAS-EXPGUI.^30,31^*R*_wp_ and *χ*^2^ fit indicators were used to assess the quality of the refined structural model.^32^ The refinements were conducted in consecutive steps as follows: background (using reciprocal interpolation), scale factors, unit cell parameters, peak width parameters, fractional atomic coordinates and (an)isotropic atomic displacement parameters. All atomic site occupancies were fixed to 1. Refinements were carried out using multiple detector banks: LiGaCl_4_ (banks 4 and 5), LiGaBr_4_ (banks 4 and 5) and LiGaI_4_ (banks 3, 4 and 5).

### Thermal analysis

The thermal stability of all samples was measured by simultaneous thermogravimetric-differential thermal analysis using a Netzsch STA 409 instrument contained within an Ar-filled MBraun UniLab recirculating glovebox (O_2_ and H_2_O < 0.1 ppm). Accurately weighed samples of 15–30 mg were heated in alumina crucibles under a constant flow of Ar (BOC, ≥99.999%, 60 mL min^−1^) from 30 °C to the desired target temperature (discussed below) at a 5 °C min^−1^ heating rate.

### Electrochemical impedance spectroscopy

Ionic conductivities were measured by AC electrochemical impedance spectroscopy (EIS) using a SP300 impedance analyser (Biologic). For LiGaCl_4_ and LiGaBr_4_, 10 mm diameter isostatically pressed pellets were obtained at room temperature by applying 325 MPa (relative densities were 79% and 71%, respectively), and were subsequently coated *via* thermal evaporation with thin Au (200 nm) electrodes. Due to the lower decomposition temperature of the iodide, Au foil was used as the electrodes. For LiGaI_4_, an uniaxially-pressed pellet was obtained at room temperature by applying 510 MPa between 10 mm diameter stainless steel rods (relative density was 90%). For LiGaCl_4_ and LiGaBr_4_, EIS analyses were conducted in the temperature range of −40 to 60 °C at frequencies from 7 MHz to 50 mHz with an excitation amplitude of 10 mV; whereas for LiGaI_4_, EIS analysis was conducted in the temperature range of −15 to 100 °C at frequencies from 7 MHz to 0.1 Hz with an excitation amplitude of 100 mV (between 50 and 100 °C) and 200 mV (between −15 and 100 °C).

### Raman spectroscopy

Raman spectra were recorded at room temperature with a Bruker VERTEX 70 spectrometer equipped with a Bruker RAM II module (1064 nm laser) with a nitrogen-cooled Ge detector. The samples were contained in sealed soda-lime glass Pasteur pipettes and measured over a region of 4000–50 cm^−1^ with a resolution of 4 cm^−1^.

### Muon-spin spectroscopy

μ^+^SR data were collected using the EMU spectrometer at the ISIS pulsed neutron and muon source, Rutherford Appleton Laboratory, UK.^33^ As with PND experiments, in order to a obtain sufficient amount of material for μ^+^SR measurements, samples were synthesised 3 times and subsequently mixed. Samples of *ca*. 1 g were loaded into titanium sample holders with a front window made of 25 μm thick titanium foil and sealed in a He-filled glovebox. Spin-polarised positive muons were implanted into the samples for a mean lifetime of 2.2 μs before decaying. The asymmetry in the count rate of the emitted positrons, *A*(*t*), was measured by two arrays of detectors on opposite sides of the sample. In order to probe the lithium diffusion behaviour in LiGaX_4_, measurements were conducted over a temperature range of 100–400 K counting 20 × 10^6^ events per run. At each temperature, measurements were made at zero applied field (ZF) and applied longitudinal fields (LF) of 10 and 20 G. A transverse field (TF) of 100 G was applied for initial asymmetry calibration. Data analysis and fitting was conducted using the Mantid open source software.^28^

## Computational details

Calculations were performed in the framework of density functional theory (DFT) within the generalised gradient approximation (GGA), using the Perdew–Burke–Ernzerhof (PBE)^34^ exchange–correlation functional as implemented in the Vienna *ab initio* simulation package (VASP).^35,36^ In these calculations, the valence electron density was expanded using a plane-wave basis set with kinetic energy cut-offs of 650, 550, and 500 eV for LiGaCl_4_, LiGaBr_4_ and LiGaI_4_, respectively. Energy convergence tests revealed negligible energy variations if the plane-waves basis set was enlarged further. The effect of the core electrons on the valence electron density was accounted for *via* the projector augmented wave (PAW) method described by Blöchl and implemented by Kresse and Joubert.^37,38^ For each of the structures the necessary numerical integrations in reciprocal space were realised using a Γ-centred Monkhorst–Pack mesh of special *k*-points, with a grid size optimised until convergence below 10^−3^ eV was achieved (6 × 6 × 3 for each of the halides).^39^ The LiGaX_4_ structures were modelled using a monoclinic unit cell (space group no. 14). To avoid any possible bias due to inaccuracies of the DFT method regarding the crystal structure, the lattice parameters of the unit cells were fixed to the experimental values obtained from the ICSD database (LiGaCl_4_, collection code 60849; LiGaBr_4_, collection code 61337; and LiGaI_4_, collection code 60850).^40^ The atomic positions were optimised using a conjugate-gradient algorithm. The structural optimization of atomic positions within the experimental unit cell was continued until the forces acting on all atoms were smaller than 10^−3^ eV Å^−1^. The threshold convergence for the electronic energy was set to 10^−5^ eV. A Gaussian smearing with a smearing width of 0.2 eV was used to enhance the convergence, although the final total energies were always extrapolated to 0 K. All calculations were performed in a not spin-polarised formalism. Dynamic stability was ensured by proper phonon calculations using the PHONOPY code.^41^ Raman spectra were computed from the above phonon calculations and the respective macroscopic dielectric tensors using the finite displacement (FD) method as implemented in VASP.^42,43^ Since the Raman active frequency values calculated by these methods are generally underestimated compared to the corresponding experimental frequencies,^44^ the former were scaled-up by a factor of 1.02 as suggested by Kesharwani *et al.*^45^

### Muon stopping site calculation

The muon stopping sites were calculated using the unperturbed electrostatic method (UEP), as implemented in the software package pymuon-suite and in the Galaxy workflow management platform,^46–50^ which has already shown good results for the modelling and interpretation of μ^+^SR experiments.^51–54^ The UEP method uses DFT calculations to estimate the host material's electrostatic potential, plus a combination of mathematical analysis and clustering techniques to estimate potential muon stopping sites. The DFT-based computer simulations carried out in this work were performed with the CASTEP^55^ code. Levels of convergence equivalent to those obtained with VASP were achieved using the same settings. Plane wave cut-offs of 650, 550, and 500 eV were used for LiGaCl_4_, LiGaBr_4_ and LiGaI_4_, respectively; and a *k*-point grid size of 6 × 6 × 3 Monkhorst–Pack was used in each case. The PBE exchange–correlation functional^23^ was also used in combination with auto-generated ultrasoft pseudopotentials.

## Results and discussion

### Crystal structures

**Fig. 1:**
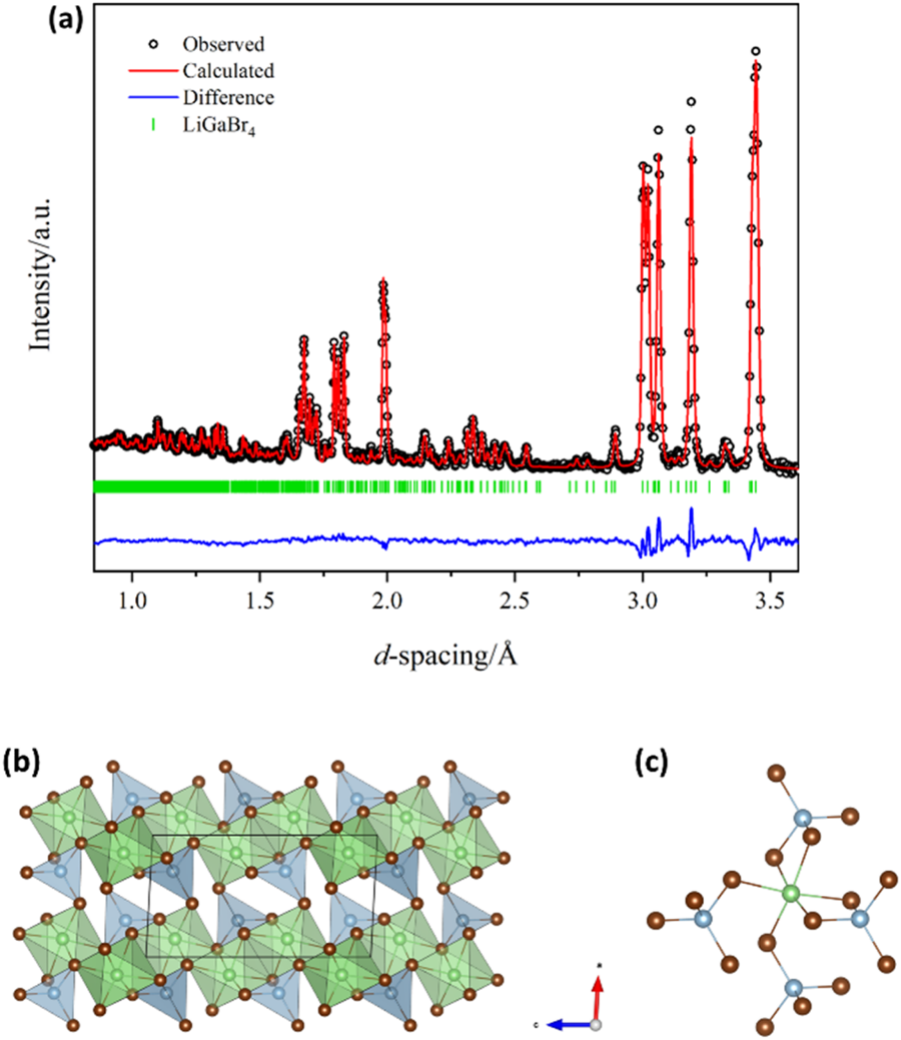
(a) Room temperature profile fit from Rietveld refinement of the structure of LiGaBr_4_ against ToF PND data (〈2*θ* = 92.59°〉 detector bank; Polaris, ISIS). *R*_wp_ = 2.44%, *χ*^2^ = 2.36. (b) Crystal structure of LiGaBr_4_ (*P*2_1_/*c*) projected in the *ac* plane as visualised with VESTA^57^ showing a polyhedral representation of the extended structure and (c) showing the linkage between an LiBr_6_ octahedron and neighbouring GaBr_4_ tetrahedra. Li, Ga, and Br atoms are represented by green, blue and brown spheres, respectively.

Although lab-based powder X-ray diffraction (PXD) provided basic structural characterisation of the tetrahalogallates (Fig. S2), which was sufficient to ascertain that single phase ternary halides had been successfully synthesised, we collected time-of-flight (ToF) powder neutron diffraction data in order to locate the Li positions accurately, allowing full characterisation of the underlying crystal chemistry of the LiGaX_4_ materials. Fig. 1 shows the profile fit for LiGaBr_4_ as an example, following Rietveld structure refinement. The respective profile fits for the chloride and iodide analogues can be found in the SI (Fig. S3–5). Crystallographic data from these refinements are collated in Tables S3–15. Lithium tetrahalogallates are isostructural to the tetrahaloaluminate analogues. Although reported crystal structures use the *P*2_1_/*a* space group setting, we have adopted *P*2_1_/*c* for consistency with our previous results.^3^ The common crystal structure of the LiGaX_4_ materials can be described in terms of a slightly distorted *hcp* X^−^ sublattice, perhaps most easily visualised along *b* axis, within which a quarter of the octahedral sites and an eighth of the tetrahedral sites are occupied by Li^+^ and Ga^3+^, respectively (Fig. 1b). The GaX_4_ tetrahedra are isolated from one another and form a sublattice similar to that seen in the SiCl_4_ structure (Fig. S6). Two LiX_6_ octahedra link across a common edge to form Li_2_X_10_ “dimeric” units. Each of these units is connected to four others by 2 axial and 2 equatorial vertices in a “*trans*” conformation that creates stepped or buckled layers that propagate in all three dimensions. Meanwhile, each GaX_4_ tetrahedron is connected to one Li_2_X_10_ unit by two edges and two other Li_2_X_10_ units by one vertex each (Fig. S7). Therefore, the extended structure can be constructed from distorted LiX_6_ octahedra and GaX_4_ tetrahedra. The octahedra are linked with four tetrahedra; two share an edge and two share a corner (Fig. 1c). As in LiAlX_4_ materials,^3^ it was found that as Cl^−^ (167 pm) is replaced by Br^−^ (182 pm) and I^−^ (206 pm),^56^ the average bond lengths increase in both the [GaX_4_]^−^ tetrahedra and the LiX_6_ octahedra, along with a concomitant linear expansion of the until cell in all three dimensions. The monoclinic distortion of the cell decreases slightly with increasing halide radius (Fig. S8). Of note, the average Li-X and Ga-X distances in LiGaX_4_ are remarkably similar to the corresponding ones in LiAlX_4_. For example, in LiAlCl_4_ the average Li–Cl and Al–Cl distances are 2.65(6) Å and 2.144(8) Å, respectively;^3^ whereas in LiGaCl_4_ the average Li–Cl and Ga–Cl distances are 2.64(3) Å and 2.177(7) Å, respectively. On the other hand, the cell parameters – and consequently the unit cell volume – as well as the polyhedral LiX_6_ volume are slightly smaller in the tetrahalogallate samples (Table S16). This contraction may influence the Li^+^ mobility by not only reducing the available diffusion pathways but also by contributing to higher activation energies (as discussed further below).

### Thermal stabilities

**Fig. 2:**
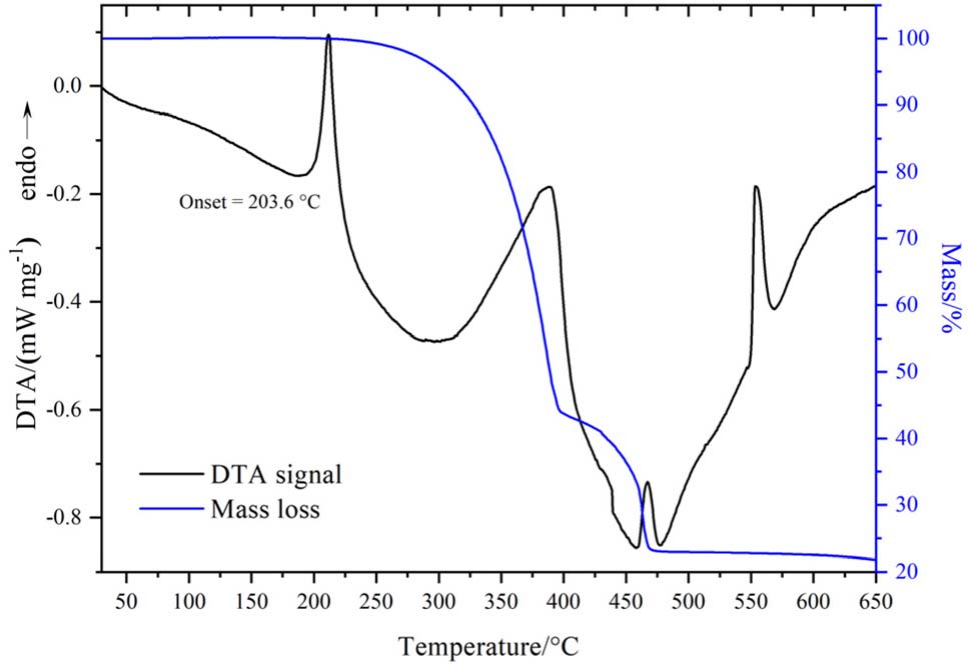
TG-DTA profiles LiGaBr_4_ heated to 650 °C at 5 °C min^−1^ under flowing Ar (60 mL min^−1^). Under these conditions LiGaBr_4_ melts at 203.6 °C followed by decomposition at *ca.* 277 °C.

In similarity to the tetrahaloaluminate analogues,^3^ the TG profiles of LiGaX_4_ (X = Cl, Br, I) are typical of thermal decomposition with volatile decomposition products (Fig. 2 and S9). For LiGaCl_4_ and LiGaBr_4_ the decomposition is preceded by melting in each case (the melting points, as determined by the respective DTA peak onsets, are summarised in Table S17), while for LiGaI_4_ the melting/decomposition processes occur simultaneously. The melting points of the tetrahalogallates increase from X = Cl (162.1 °C) through Br (203.6 °C) to I (251.0 °C) and were found to be *ca.* 15 °C higher than their respective LiAlX_4_ analogues.^3^ Unlike LiGaCl_4_ and LiGaI_4_, LiGaBr_4_ decomposes *via* a two-step process. The first step begins at approximately 250 °C and concludes around 400 °C, resulting in a mass loss of 56.28%, whereas the second step beings around 425 °C and ends near 470 °C. The total mass across both steps is 79.02%. An inspection of the STA profiles towards the end of the heating processes indicates that the respective LiX phases remain in the final products after decomposition, since the onset temperatures of each of the high temperature endothermic DTA peaks agrees with the reported melting points for the relevant binary LiX salts.^58^ These observations are consistent with the experimental mass losses (wt%) for the decomposition mechanism LiGaX_4_ → GaX_3_ + LiX (*i.e.* assuming that all GaX_3_ is lost as a gas, Table S18). Nevertheless, mass spectrometric analysis of the evolved gases would be required for unambiguous identification of the decomposition products and further validation of the proposed mechanism. TG-DTA also confirmed the absence of unreacted GaX_3_ in the synthesised LiGaX_4_ materials with no GaX_3_ melting transitions visible in the DTA data (*e.g.* GaCl_3_ melts at 77.9 °C).^58^

### Electronic structure

**Fig. 3:**
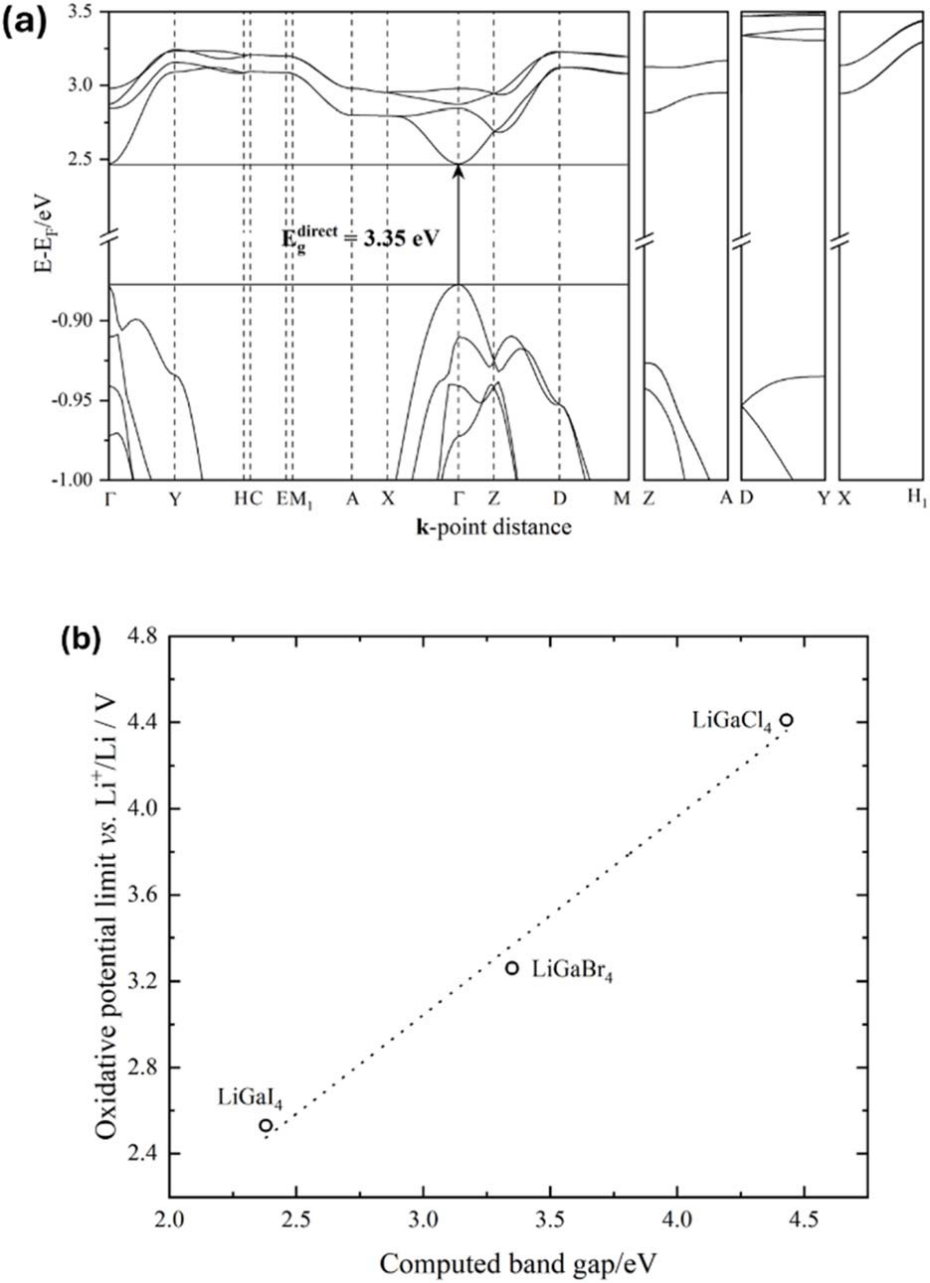
(a) Computed (DFT/PBE) band structure of LiGaBr_4_ with band energies scaled to the Fermi level (*E*_F_). (b) Correlation between computed oxidative potential limit^12^ and computed band gap energies of LiGaX_4_ (X = Cl, Br, I) materials. The dotted line corresponds to a linear fit (*R*^2^ = 0.991).

Compared to tetrahaloaluminate analogues,^59^ one could expect lower band gap values in LiGaX_4_ materials. This does not mean that the upper limit of the electrochemical stability window (EW) of tetrahalogallates will also be lower since this value is determined by the oxidation potential of X = halogen. The DFT calculations carried out at the equilibrium geometry, using the PBE exchange–correlation functional, show that LiGaCl_4_ and LiGaBr_4_ are wide direct band gap materials with, possibly underestimated, values of 4.43 eV and 3.35 eV, respectively, while LiGaI_4_ was found to have an indirect gap along the Γ → X line with a value of 2.38 eV (Fig. 3a and S10). In similarity with the LiAlX_4_ materials, the covalent character between Li^+^ and complex [GaX_4_]^−^ bonds increases from Cl < Br < I, which is characterised by a decrease in the anion p-band centres with respect to the mid-gap^60^ and it was also found to correlate with the computed oxidative potential limits available in the literature^12^ (Fig. S11 and 12). This is also consistent with the fact that the computed oxidative potential increases when increasing the band gap (Fig. 3b). Due to the highly hygroscopic/deliquescent nature of lithium tetrahalogallates,^24^ it was not possible to successfully perform DR-UV-Vis measurements. However, a plot of computed EWs available in the literature,^12,61^ including the tetrahaloaluminates, *versus* the computed band gaps confirmed the hypothesis that the above quantities are linearly correlated across the LiMX_4_ halometalates (Fig. S13).

### Raman spectra

Raman spectra of LiGaX_4_ were measured to follow the progress of the reaction and to confirm the composition, structure and bonding of the products. In this work, the normal modes of vibration in crystals were obtained by nuclear site group analysis.^62^ Since LiMX_4_ materials (M = Al, Ga; X = Cl, Br, I) are isostructural, the optical vibrational modes for lithium tetrahalogallates are given by eqn (1).^59^

1 Γ_optic_ = 18A_g_ (R) + 17A_u_ (IR) + 18B_g_ (R) + 16B_u_ (IR)

**Fig. 4:**
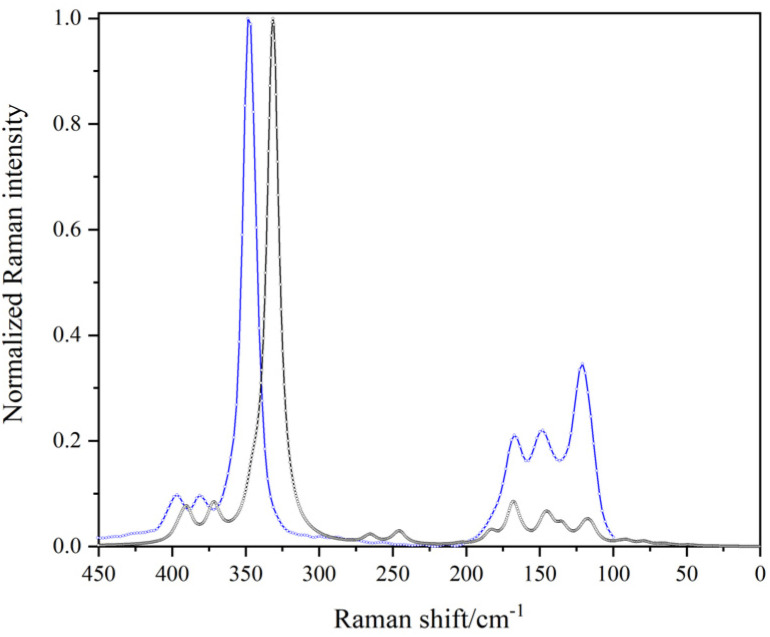
Theoretically simulated (DFT/FD/PBE; black line) *vs.* experimentally observed (blue line) Raman spectrum of LiGaCl_4_ (*λ*_laser_ = 1064 nm).

Table S19 summarises all the Raman frequencies by the finite displacements (FD) method. Although 36 Raman active modes are predicted, some of the bands were found to be very low in intensity and cannot be easily observed experimentally (Fig. S14–16). For LiGaCl_4_ there is a good agreement between the measured and calculated Raman spectra within the harmonic approximation (Fig. 4). Conversely, for the bromide and iodide equivalents the agreement is less evident (Fig. S15 and 16). The experimental Raman spectra show that any vibrations that may have been attributed to starting materials, *i.e.* GaX_3_ (Raman active) were not present, in agreement with the diffraction and thermal data, which served to corroborate the reaction completion and high purity of the final products (Fig. S17). The Raman shifts and their corresponding modes of vibration for GaX_3_ materials can be found in the SI (Fig. S18 and Table S20).

### Macroscopic ion transport

**Fig. 5:**
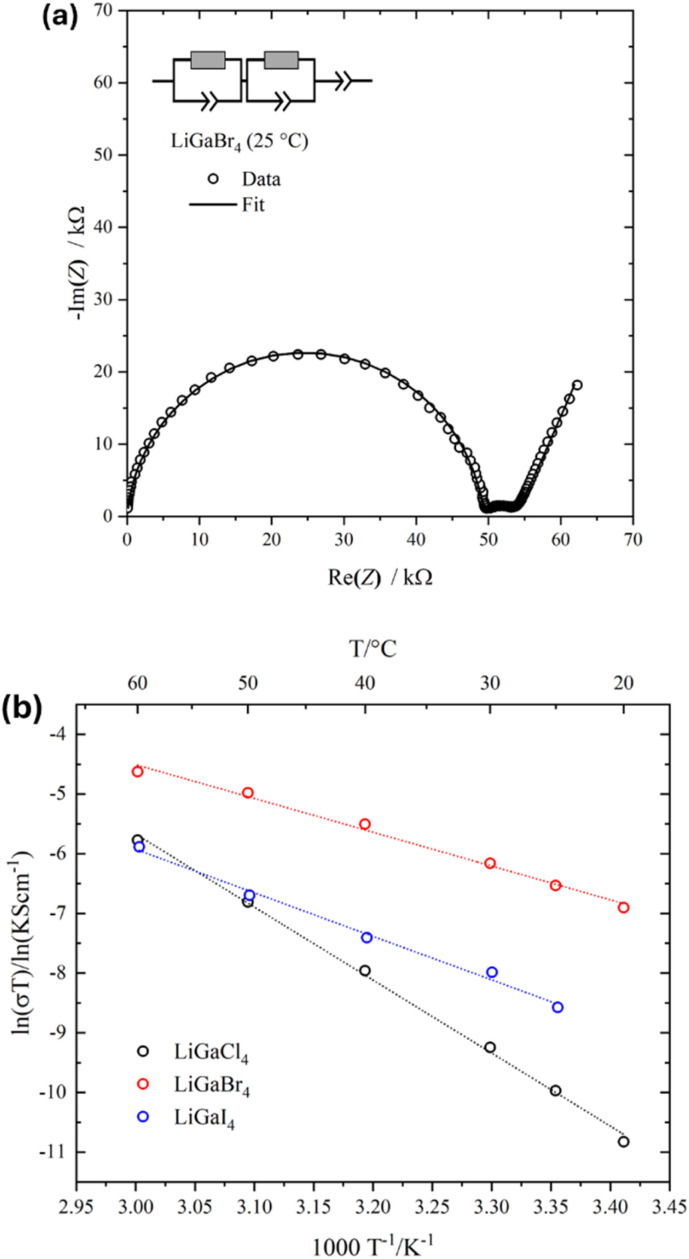
(a) Room temperature Nyquist plots of LiGaBr_4_, showing the impedance responses (open circles) and fits (solid lines). The geometric density is 71%. (b) Arrhenius plots of conductivity values obtained from temperature-dependent impedance spectroscopy for LiGaX_4_ materials (X = Cl, Br, I).

Kahle *et al.*^26^ identified LiGaI_4_ as a potential fast-ionic conductor based on full first-principles molecular dynamics calculations performed at simulated temperatures over the temperature range 500–1000 K (227–727 °C), obtaining an activation energy of 0.35 ± 0.06 eV. However, our experimental thermal analysis shows that the iodide has completely decomposed to LiI(s) + GaI_3_(g) at 380 °C. Fig. 5a shows the room temperature Nyquist plot of LiGaBr_4_. The respective plots for the chloride and iodide are shown in Fig. S19. Due to the high resistivity of the samples, responses at lower temperatures (*i.e.* below 10 °C) produced poor quality plots, from which no meaningful capacitances or resistances could be extracted. For X = Cl and Br, the spectra were fitted with an equivalent circuit consisting of two parallel constant phase elements (CPE)/resistors in series with a further CPE. At 25 °C, the capacitances of the high-frequency CPE/resistor elements are 1.9 × 10^−11^ (LiGaCl_4_) and 2.3 × 10^−11^ F (LiGaBr_4_) with *α*-values of 0.93 and 0.95 respectively, which represent the ideality of the CPE. At lower frequencies, impedance contributions are observed with capacitances of 1.6 × 10^−8^ F (X = Cl) and 3.6 × 10^−7^ (X = Br). This can be attributed to a surface layer, which may be formed on thermal decomposition during gold coating, for example.^11,63^ On the other hand, the spectrum from the LiGaI_4_ pellet was fitted with an equivalent circuit consisting of one parallel CPE/resistor in series with a further CPE. The capacitance of the CPE/resistor is 5.0. × 10^−11^ F with an *α*-value of 0.98. We note that capacitances for LiGaX_4_ samples are in the 10^−11^ F range, indicating that either ordered bulk or disordered grain boundary (GB) contributions cannot be excluded. Therefore, the obtained resistance values were evaluated to total ionic conductivities.^63,64^ It is worth noting that, due to the lower thermal stability of LiGaI_4_, Au foil was used instead of thermally evaporated electrodes, and a higher uniaxial pressure was applied to ensure good interfacial contact.

Arrhenius behaviour was noted in the LiGaX_4_ series across a temperature range of 20–60 °C for X = Cl and Br, and 25–60 °C for X = I (Fig. 5b). For the latter, the data collected over 60 °C may indicate a change in the conduction mechanism (Fig. S20). Room temperature total ionic conductivities (*σ*_RT_) and the extracted activation energies from linear fits of the Arrhenius plots (*E*_a_) are summarised in Table S21. The measured ionic conductivity for LiGaBr_4_ agrees with the value reported by Tomita *et al.* (7 × 10^−6^ at 24 °C),^24^ but is lower than the recent reported value by Gao *et al.* (2.0 × 10^−5^ at 25 °C),^25^ which may be attributed to the specific experimental parameters and the setup used in each measurement, such as the relative density of the pellet, pressure under measurement, *etc.*^65,66^ It is also worth noting that this is the only compound in the LiGaX_4_ series for which Li^+^ conductivity has previously been reported, and that the deliquescent nature of the halogallates has hindered the determination of their bulk properties.^24^ In fact, Tomita *et al.* reported that the ionic conductivity of LiGaBr_4_ was measured only at room temperature due to its strong hygroscopic character.^24^ The relatively high activation energy for LiGaCl_4_ obtained by EIS suggests that, in addition to ion migration, other contributions such as defect formation enthalpies and GB resistance may play a significant role in the total resistance. For example, Liu *et al.*^67^ found that Li_3_YBr_3_Cl_3_ pellets prepared by hot pressing, compared to those made by cold pressing, exhibited improved GB contact and reduced GB resistance, both of which contributed to enhanced overall ionic conductivity. Additionally, due to the deliquescent nature of tetrahalogallates, the water content in glovebox atmospheres and during measurements may affect their chemical stability. These considerations are consistent with the fact that the measured activation energy for LiGaBr_4_*via* EIS is much higher than the value obtained by a local probe such as ^7^Li NMR, *i.e.* 0.49 eV *vs.* 0.37 eV, respectively.^24^ In this regard, a more detailed study of the microstructure of LiGaX_4_ is needed to help understand the cause for the differences in the extracted activation energies, especially for X = Cl. In addition, further studies of the sample preparation procedures, *i.e.* densification procedure, pelletising pressure, applied pressure during measurement and pellet contacting method,^65^ are required to obtain the ‘true’ ionic conductivity values across the tetrahalogallates.

### Microscopic ion transport probed by μ^+^SR

μ^+^SR experiments were conducted to study the local ionic dynamics in LiGaX_4_, which seem to be greatly affected by additional contributions as shown by EIS. In 2000, Tomita *et al.*^24^ used temperature-dependent ^7^Li NMR to estimate the activation energy of Li^+^ diffusion in LiGaBr_4_ reporting a value of 370 meV. Since then, no further studies on the local Li^+^ dynamics have been conducted on any of the other tetrahalogallates. In this work, the acquired data for LiGaX_4_ materials were fitted simultaneously by a combination of a dynamic Gaussian Kubo-Toyabe function, DGKT (*Δ*, *ν*, *t*), to describe the Li^+^ diffusion; an exponential muon decay function, *A*_μ_ e^−*λt*^, to describe the muonium formation; and a flat background (BG) signal, to account for the fraction of muons stopped in the sample holder, as follows:

2 *A*_0_*P*(*t*) = *A*_KT_*G*^DGKT^ (*Δ*, *ν*, *t*) +*A*_KT_ e^−*μt*^ + *A*_BG_

where *A*_0_ is the initial asymmetry, *i.e.* at *t* = 0; and *A*_KT_, *A*_μ_ and *A*_BG_ are the asymmetries associated with the three signals. *Δ* is the static width of the local field distribution at the muon site, *ν* is the field fluctuation rate, and *λ* is the muon decay rate.

**Fig. 6:**
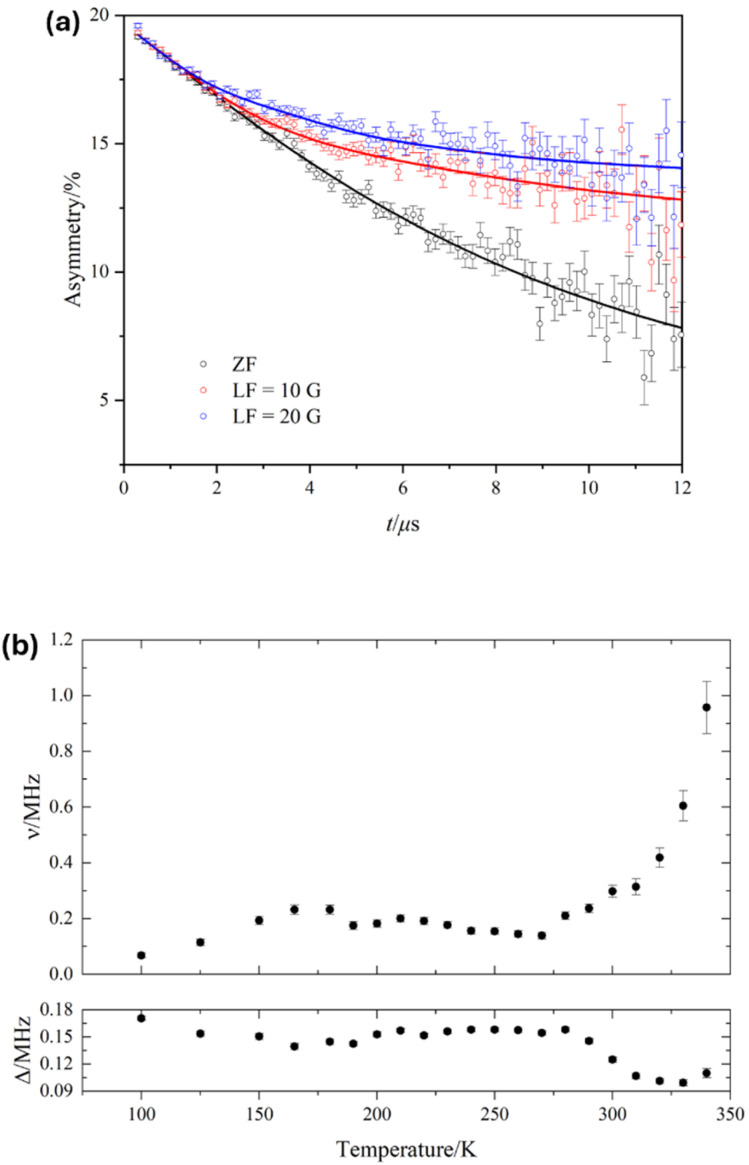
(a) ZF and two LF (10 and 20 G) μ^+^SR spectra for LiGaBr_4_ at 300 K, showing data points (open circles) and fits (solids lines) using eqn (2). (b) Temperature dependences of the (top) fluctuation rate, *ν*, and (bottom) static widths of the local field distribution, *Δ*, using eqn (2).

**Fig. 7:**
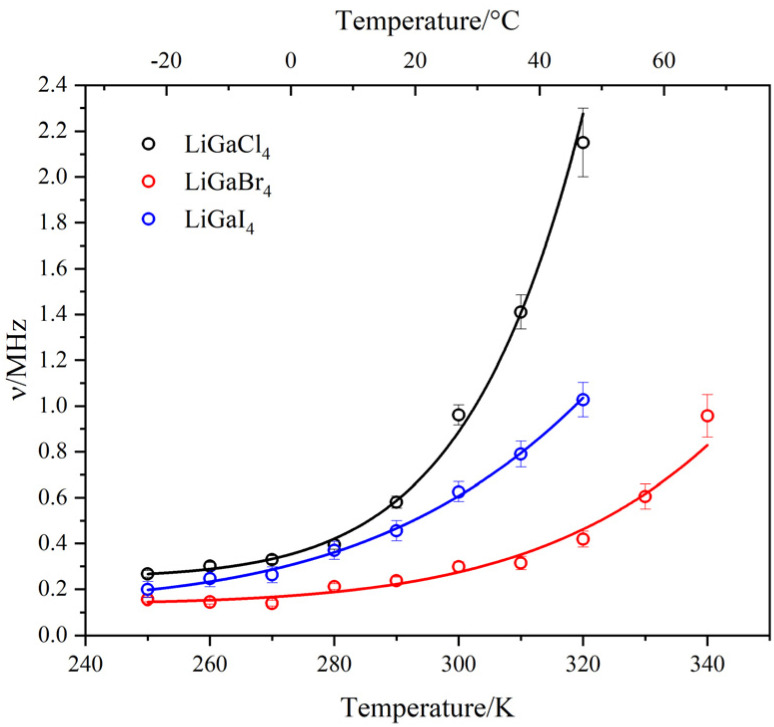
Temperature dependence of the field fluctuation rate (*ν*) in LiGaX_4_ materials (X = Cl, Br, I) fitted by eqn (3) (solid lines) from μ^+^SR data (open circles).

For X = Cl and Br, the *A*_KT_ and *A*_BG_ were obtained by fitting the data collected at 100 K and kept fixed throughout the analysis (*A*_BG_ = 0.033 and *A*_KT_ = 0.16 for X = Cl; *A*_BG_ = 0.028 and *A*_KT_ = 0.116 for X = Br). For X = I, *A*_BG_ was fixed to 0.054 from 150 K upwards while *A*_KT_ was allowed to vary freely to obtain the best fits (See Fig. 6a as an example). The temperature dependencies of both *Δ* and *ν* are shown in Fig. 6b. Generally speaking, *ν* is characterised by a nearly constant region in the lower temperature range, followed by an exponential increase starting at *ca* 250 K (−23 °C) that is explained by a thermally activated process, attributed to diffusive motion of either Li^+^ or μ^+^. Since *Δ* remains nearly constant up to 250 K, it can be assumed that the muon is static in the crystal lattice, so the diffusion can be attributed to Li^+^ only. Furthermore, the calculated stable stopping sites for μ^+^ are all in the vicinity of the X^−^ (X = Cl, Br, I. See Fig. S21). The calculated bond distances for the μ^+^–X^−^ bond are: 1.33 Å, 1.50 Å, and 1.75 Å, respectively. These distances agree well with the experimental values for the molecular equivalent bond lengths; 1.28 Å, 1.41 Å, and 1.61 Å for H^+^–X^−^ (X = Cl, Br, I).^68^ This means that the μ^+^–X^−^ bonds in LiGaX_4_ are stable, and that, for *T* < 350 K, the μ^+^ is likely to remain in its stopping site while Li^+^ diffuses. For example, in typical cathode materials, the evidence shows that muons remain static with energy barriers for diffusion which are larger than the ion that is known to be mobile from the bulk properties.^69^ At temperatures >300 K, the value of *Δ* decreases as Li^+^ becomes more dynamic. Increased Li^+^ mobility leads to rapid fluctuations of the local nuclear dipolar fields at the muon site, effectively averaging out the static field distribution and resulting in a reduced *Δ*. The increase (rather than a decrease) in *Δ* observed for LiGaCl_4_ and LiGaI_4_ can be explained by the difficulty of extracting the KT parameters when approaching the *Δ* ≤ 0.1*ν* limit (Fig. S22).^70^ The activation energies attributed to Li^+^ diffusion were extracted by fitting *ν*(*T*) to an Arrhenius-like equation,

3 *ν*(*T*) = *A* + *B* exp(−*E*_a_/*k*_B_*T*),

with values of 479 ± 33, 352 ± 64 and 268 ± 23 meV for X = Cl, Br and I, respectively (Fig. 7). The extracted activation energy for Li^+^ diffusion in LiGaBr_4_ is in good agreement with the reported value from NMR measurements, *i.e.* 352(64) *vs.* 370 meV, which serves to corroborate the hypothesis that macroscopic values of conductivity are influenced by additional contributions, and that softer lattices (*i.e.* increasing lattice polarizability as a function of X = Cl, Br, I) provide lower activation energies for Li^+^ migration in isostructural compounds.^71^ It is worth pointing out that these activation energies are higher than other Li^+^ battery materials studied by μ^+^SR,^72^ which suggests that the tetrahalogallates are intrinsically unexceptional Li^+^ conductors. Finally, we used the fluctuation rates attributed to Li^+^ to obtain the diffusion coefficients (*D*_Li_) in LiGaX_4_, according to eqn (4),4
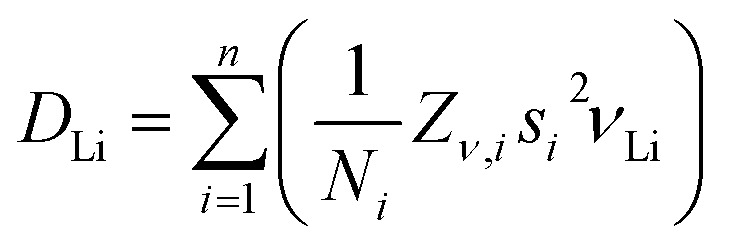
where *N*_*i*_ is the number of Li^+^ sites in the *i*th path, *Z*_*v*,*i*_ is the vacancy fraction, and *s*_*i*_ is the jump distance. According to our migration pathway analysis performed using SoftBV (see below), in LiGaBr_4_ (used here as an example) Li^+^ can diffuse in either direction along a 1D channel (to ‘i5’ and ‘i4’ sites), *i.e.*, *n* = 2 (Fig. S23). Since the number of vacant sites in each direction is one, *N*_1_ = *N*_2_ = 1; and these are empty, thus, *Z*_1_ = *Z*_2_ = 1. The jump distances between Li^+^–i4 and Li^+^–i5 are 2.50 and 2.41 Å, respectively. At 300 K, *ν* = 0.30 MHz, therefore we estimated *D*_Li_ ∼3.6 × 10^−10^ cm^2^ s^−1^. The corresponding values for X = Cl and I are ∼1.1 × 10–9 cm^2^ s^−1^ and ∼8.8 × 10–10 cm^2^ s^−1^, respectively. Although LiGaCl_4_ exhibits relatively fast local dynamics, as indicated by its *D*_Li_, it simultaneously shows the lowest Li^+^ conductivity across the series. This discrepancy between short- and long-range transport suggests that rapid local Li^+^ hopping does not necessarily translate into high bulk conductivity. While factors such as grain boundaries, porosity, and relative pellet density can influence long-range conduction,^72,73^ the conductivity trend observed in LiGaX_4_ cannot be explained by density alone. Instead, differences in activation barriers, defect formation enthalpies, and percolation connectivity likely play a more dominant role in limiting macroscopic transport. This observation aligns with the fact that *E*_a_ values derived from μ^+^SR are significantly lower than those obtained from EIS, implying the presence of additional energy barriers that hinder long-range diffusion. It is important to note that *D*_Li_ values from μ^+^SR generally reflect local hopping rates rather than long-range transport. In LiGaCl_4_, although Li^+^ ions move rapidly between atomic sites, sustained diffusion over longer distances may require higher energy (see below).

### Conduction mechanisms

**Fig. 8:**
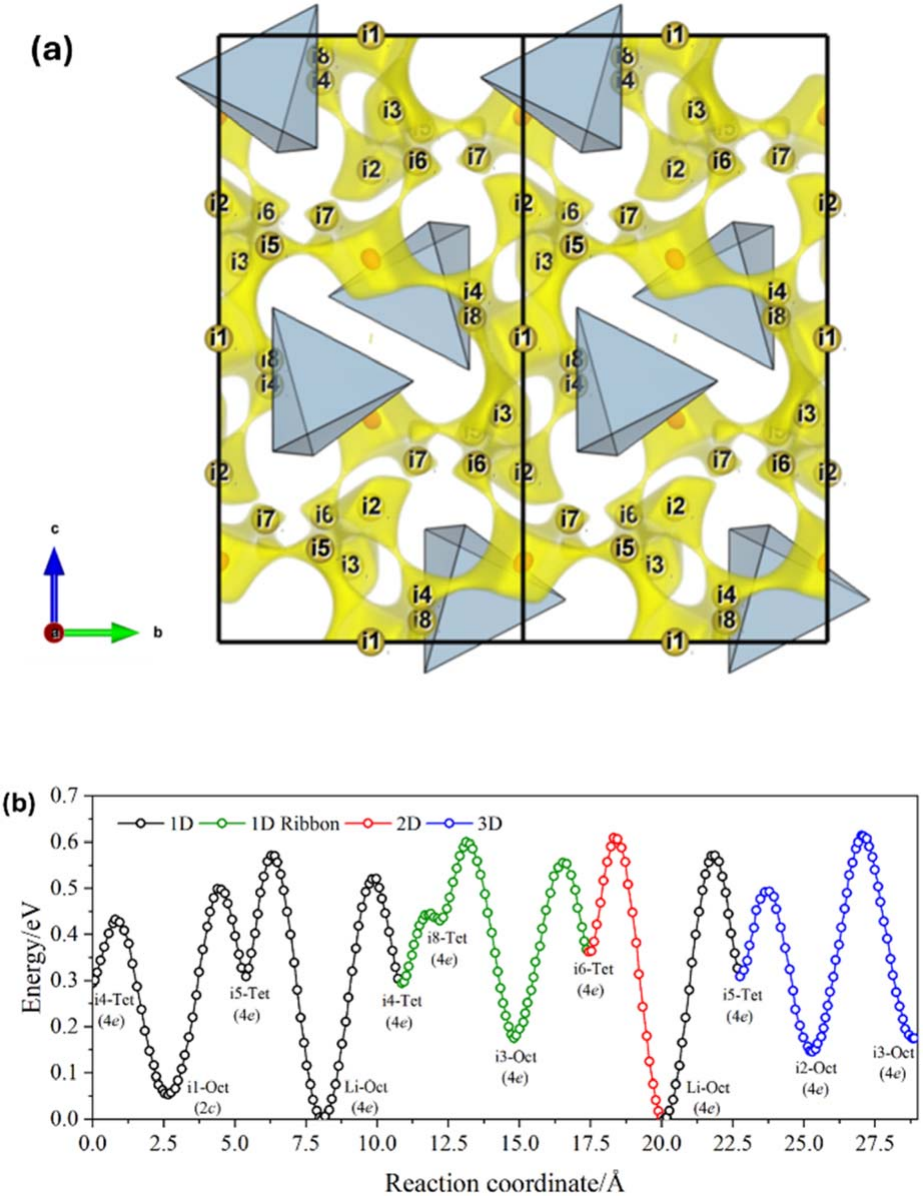
(a) BVSE map showing Li migration pathways in a (100) projection of the LiGaBr_4_ structure, as visualised with VESTA.^57^ The highest isosurface level of 0.62 eV over the global minimum is shown in yellow. Red dots indicate octahedral Li lattice sites and yellow spheres indicate tetrahedral/octahedral interstitial sites. (b) BVSE models of migration barriers for LiGaBr_4_ derived reference data (ICSD, collection code 61337).^40^ The relative site energy is zero for Li lattice sites.

The possible Li^+^ diffusion pathways in the tetrahalogallates were obtained *via* bond-valence site energy (BVSE) analysis as implemented in the SoftBV package.^74^ Unlike the Al-doped halides equivalents,^3^ the migration pathways in the tetrahalogallates appear to have a lower dimensionality, which are dominated by 1D jumps. This difference may attributed to the reduced available space for Li^+^ diffusion, as both the unit cell and LiX_6_ polyhedral volumes decrease in the Ga-doped equivalents. Additionally, since Al is more electronegative than Ga, a lower charge density on the halide sites is expected in LiAlX_4_. As a result, the coulombic interaction between Li^+^ and X^−^ becomes weaker, thereby lowering the barrier for Li^+^ migration – a phenomenon referred to as the inductive effect.^73^ Based on this rationale, LiGaX_4_ are expected to exhibit inherently higher activation energies for Li^+^ diffusion. The BVSE maps of LiGaX_4_ are presented in Fig. 8 and S24. By using LiGaBr_4_ as an example to describe the conduction mechanisms, the bottleneck for 1D conduction along the [010] vector involves Li^+^ hopping from its normal octahedral 4e lattice position to an adjacent tetrahedral 4e site (‘i5’ in the BVSE map notation). In addition, two tetrahedral 4e sites are available for Li to occupy: ‘i4’ and ‘i7’, although the latter is not included in the recommended migration pathway suggested by SoftBV. The 1D ribbon path is defined as a path that percolates in only one dimension, but its percolating nature cannot be eliminated by removing any of the segments that build the path. This is relevant when immobile atoms (*e.g.* dopants) are added to the structure, because 1D ribbon paths are less vulnerable against blocking compared to the normal 1D path. On the other hand, the bottleneck for 2D conduction along the (100) plane would involve Li^+^ hopping from its normal octahedral 4e lattice to an adjacent tetrahedral 4e site (‘i6’). Alternatively, 3D conduction would require either a jump from tetrahedral 4e to octahedral 4e sites (‘i5’ → ‘i2’) or from octahedral 4e to octahedral 4e sites (‘i2’ → ‘i3’). As in LiAlX_4_, replacing X = Cl for X = Br and I stabilises the octahedral 2a (2c) site as its relative site energy decreases. Generally speaking, the BVSE models of the migration barriers in LiGaBr_4_ and LiGaI_4_ depict similar energy landscapes and indicate that the conduction pathways do not change significantly upon halide substitution. On the other hand, LiGaCl_4_ shows fewer 1D ribbon-like channels compared to the bromide and iodide analogues (Fig. 8a, S24b and d). This limited connectivity may increase the vulnerability to blocking effects limiting macroscopic conductivity. Nonetheless, the differences in ionic conductivity across the series likely result from a combination of factors, rather than channel topology alone. In addition, further studies, *e.g.* MD simulations, should be performed to evaluate the effect of anion dynamic disorder on Li^+^ transport, such us the libration/reorientation of [GaX_4_]^−^ units, as observed for analogous [AlX_4_]^−^ anions in LiAlCl_4_.^3,75^

## Conclusions

In this work, we have demonstrated that high purity lithium tetrahalogallate powders can be easily synthesised *via* mechanochemical methods. Nevertheless, the characterisation of these Ga-containing halides is challenging due to their deliquescent nature. Compared to the Al-containing equivalents, LiGaX_4_ materials have lower thermal stabilities (*i.e.* decompose at lower temperatures)*.* This is particularly problematic for EIS measurements conducted on pellets coated *via* thermal evaporation with gold electrodes. Further studies of the sample preparation procedures, *i.e.* densification procedure, pelletising pressure, applied pressure during measurement and pellet contacting method, are required to obtain the ‘true’ ionic conductivity values across the tetrahalogallates.

From macro- and microscopic transport measurements, it is clear that additional phenomena have an impact on the ionic conductivities of LiGaX_4_ materials, especially for X = Cl. Additional investigations are required to evaluate the effects of grain boundaries and moisture in Ga-containing halides, and also how these can be mitigated. Preliminary results indicate that LiGaBr_4_ has the highest ionic conductivity at room temperature (4.87 × 10^−6^ S cm^−1^) among the series. Compared to LiAlX_4_, the diffusion pathways in LiGaX_4_ showed a lower dimensionality and higher activation energies for Li^+^ diffusion, which would lead to lower ionic conductivities.

Despite a common monoclinic structure, the LiGaX_4_ series (X = Cl, Br, I) exhibits markedly different ionic conductivities, with LiGaCl_4_ being the lowest. μ^+^SR measurements reveal rapid local Li^+^ hopping in LiGaCl_4_ but the highest activation energy for long-range diffusion, demonstrating that fast local motion does not guarantee macroscopic conduction. BVSE calculations show fewer, less interconnected 1D pathways in LiGaCl_4_, increasing susceptibility to blocking. Additional microstructural factors, such as pellet density, porosity, and grain boundaries may further hinder long-range transport, explaining the discrepancy between microscopic and bulk measurements.

Finally, DFT calculations indicate a general correlation between computed band gaps and EWs in LiMX_4_ materials (M = Al, Ga; X = Cl, Br, I). From μ^+^SR data, it was demonstrated that that softer lattices provide lower activation energies for Li^+^ migration. Collectively, these results highlight the complex interplay between lattice dynamics, microstructure, and ionic transport in LiGaX_4_ halides.

## Author contributions

Nicolás Flores-González, conceptualisation, supervision, funding acquisition, writing – original draft, review & editing; Martí López, formal analysis, investigation, writing – review & editing; Nicolò Minafra, formal analysis, investigation, writing – review & editing; Jamie Jack, investigation; Jan Bohnenberger, investigation; Atsushi Inoishi, formal analysis, investigation, writing – review & editing; Nalin Gupta, formal analysis, investigation, writing – review & editing; Leandro Loborio, formal analysis, investigation, writing – review & editing; Francesc Viñes, supervision, writing – review & editing; Ronald I. Smith, formal analysis, investigation, writing – review & editing; Peter J. Baker, formal analysis, investigation, writing – review & editing; Ingo Krossing, investigation, writing – review & editing, Wolfgang G. Zeier, supervision, funding acquisition, resources, writing – review & editing; Francesc Illas, supervision, funding acquisition, resources, writing – review & editing; and Duncan H. Gregory, conceptualisation, supervision, funding acquisition, resources, writing – review & editing.

## Conflicts of interest

There are no conflicts to declare.

## Supplementary Material

SC-OLF-D5SC03999A-s001

## Data Availability

Data collected on the Polaris and EMU instruments at the ISIS Pulsed Neutron and Muon Source (proposals XB1990132, 1990133, 1990134 and RB2010580) are available from: https://doi.org/10.5286/ISIS.E.RB1990132-1, https://doi.org/10.5286/ISIS.E.RB1990133-1, https://doi.org/10.5286/ISIS.E.RB1990134-1 and https://doi.org/10.5286/ISIS.E.RB2010580-1. Additional data supporting this article have been included in the supplementary information (SI). Supplementary information: experimental procedures, crystallographic information, TG-DTA profiles, electronic structure, Raman spectra, macro- and microscopic ion-transport analyses, muon stopping sites, jump distances, and BVSE analysis. See DOI: https://doi.org/10.1039/d5sc03999a.
