# Supplementary material for: Insight into the prospects and limitations of mechanochemically-synthesised lithium tetrahalogallates, LiGaX4 (X = Cl, Br, I), as Li-ion conductors
Source: Chem Sci. 2025 Nov 6;16(48):23299–309. doi: 10.1039/d5sc03999a (PMC12590958; doi:10.1039/d5sc03999a)
Supplement: SC-016-D5SC03999A-s001 [file SC-016-D5SC03999A-s001.pdf]

Supplementary Information (SI) for Chemical Science.
This journal is © The Royal Society of Chemistry 2025

Insight into the prospects and limitations of mechanochemically-synthesised lithium tetrahalogallates, $LiGaX_4$ (X = Cl, Br, I), as Li-ion conductors

*Nicolás Flores-González, Martí López, Nicolò Minafra, Jamie Jack, Jan Bohnenberger, Atsushi Inoishi, Nalin Gupta, Leandro Liborio, Francesc Viñes, Ronald I. Smith, Peter J. Baker, Ingo Krossing, Wolfgang G. Zeier, Francesc Illas and Duncan H. Gregory**

Dr. Nicolás Flores-González, Jamie Jack, Prof. Duncan H. Gregory
School of Chemistry, Joseph Black Building
University of Glasgow
Glasgow, G12 8QQ, United Kingdom
E-mail: duncan.gregory@glasgow.ac.uk

Dr. Nicolás Flores-González
Department of Materials Engineering
Faculty of Engineering
University of Concepción
Concepción 4070415, Chile

Dr. Martí López, Dr. Francesc Viñes, Prof. Francesc Illas
Departament de Ciència de Materials i Química Física
Institut de Química Teòrica i Computacional (IQTCUB)
Universitat de Barcelona
c/Martí i Franquès 1-11, 08028 Barcelona, Spain

Dr. Nicolò Minafra, Prof. Wolfgang G. Zeier
Institute for Inorganic and Analytical Chemistry
University of Münster
Correnstr. 39, 48149, Germany

Prof. Wolfgang G. Zeier
Institute of Energy Materials and Devices (IMD)
Helmholtz-Institut Münster (IMD-4: HI MS)
Forschungszentrum Jülich
Münster D-48149, Germany

Dr. Jan Bohnenberger, Prof. Ingo Krossing
Institut für Anorganische und Analytische Chemie
Freiburger Materialforschungszentrum (FMF)
Universität Freiburg
Albertstr. 21, 79104 Freiburg, Germany

Nalin Gupta, Dr. Ronald I. Smith, Dr. Peter J. Baker, Dr. Leandro Liborio
ISIS Pulsed Neutron and Muon Source
STFC Rutherford Appleton Laboratory
Didcot, Oxfordshire, OX11 0QX, United Kingdom

Dr. Atsushi Inoishi
Institute for Materials Chemistry and Engineering
Kyushu University
Kasuga-koen 6-1, Kasuga, Fukuoka, 816-8580 Japan

Synthesis

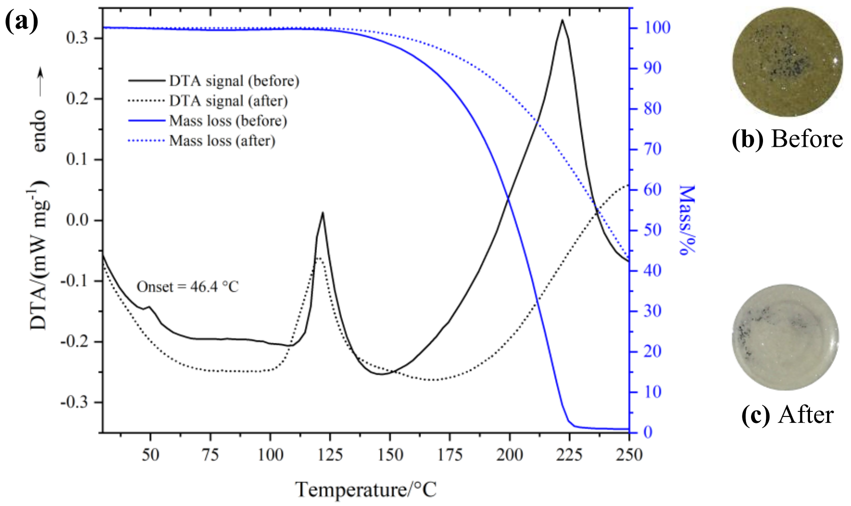

Figure S1. (a) STA analysis of commercial $GaBr_3$ (Alfa Aesar, anhydrous, ≥ 99%). The visual difference between pre-and post-sublimated samples is shown in **(b)** and **(c)**, respectively.

Table S1. Experimental parameters for the synthesis of $LiGaX_4$ (X = Cl, Br, I).

Reagents	N° balls	Speed / rpm	Milling time / h	Ball:powder ratio
1:1 $LiCl:GaCl_3$	10	200	3	80:1
1:1 $LiBr:GaBr_3$	10	300	8	80:1
1:1 $LiI:GaI_3$	10	200	6	80:1

Table S2. Colours of as-synthesised $LiGaX_4$ (X = Cl, Br, I).

Composition	Colour
$LiGaCl_4$	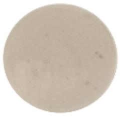
$LiGaBr_4$	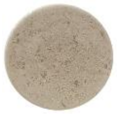
$LiGaI_4$	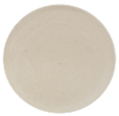

Powder X-Ray diffraction (PXD)

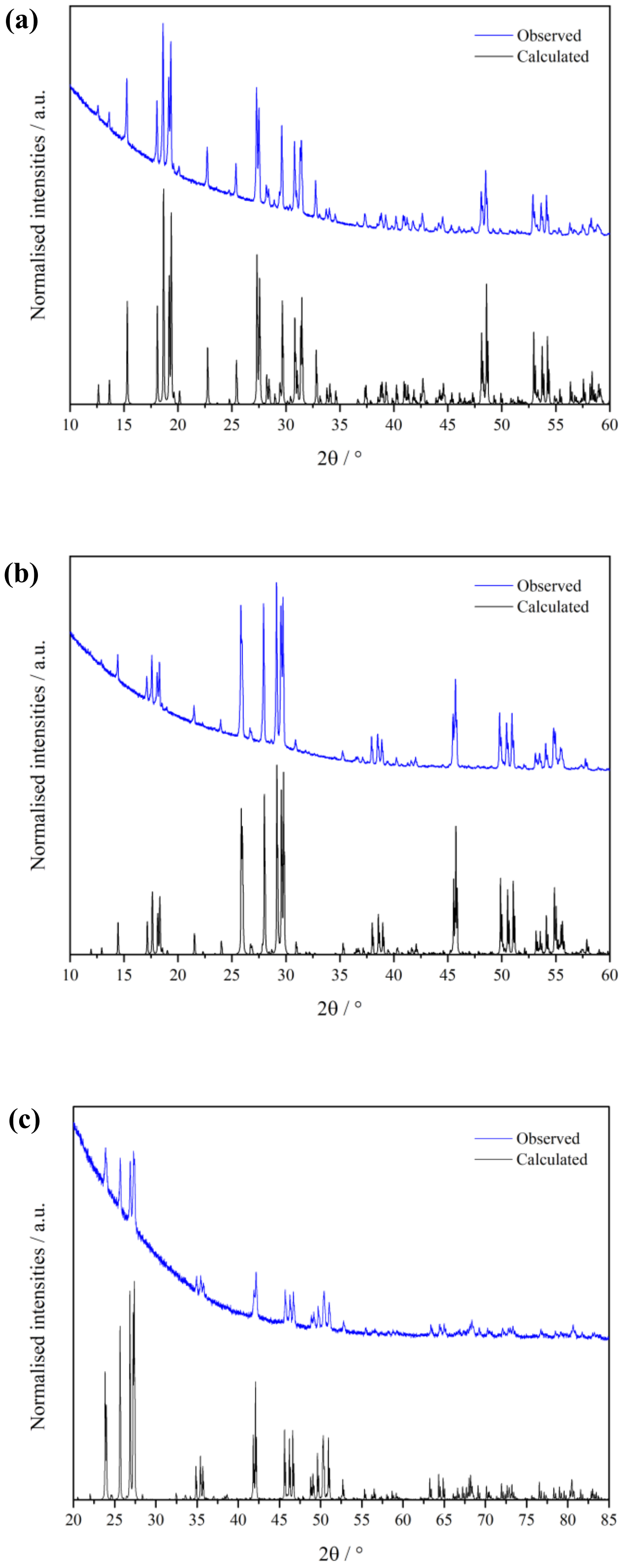

Figure S2. Room temperature PXD patterns of mechanochemically-synthesised **(a)** $LiGaCl_4$, **(b)** $LiGaBr_4$ and **(c)** $LiGaI_4$, as compared with simulated diffraction patterns calculated using the structures reported in the ICSD database ($LiGaCl_4$, collection code 60849; $LiGaBr_4$ collection code 61337; and $LiGaI_4$, collection code 60850).

Crystal structures

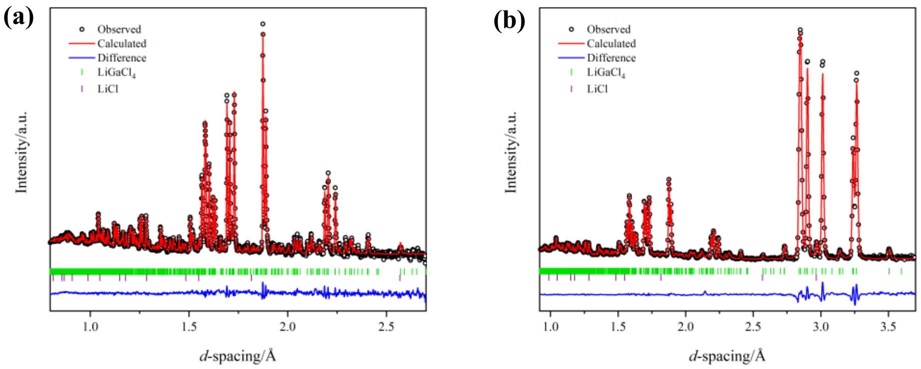

Figure S3. Room temperature profile fits from Rietveld refinement against time-of-flight powder neutron diffraction data for $LiGaCl_4$, collected at Polaris, ISIS. **(a)** detector bank 5, ($\langle 2\theta = 146.72°\rangle$), R_{wp} = 1.79 %, R_p = 2.52 %; **(b)** detector bank 4, ($\langle 2\theta = 92.59°\rangle$). R_{wp} = 1.36 %, R_p = 2.59 %, χ^2 = 1.91.

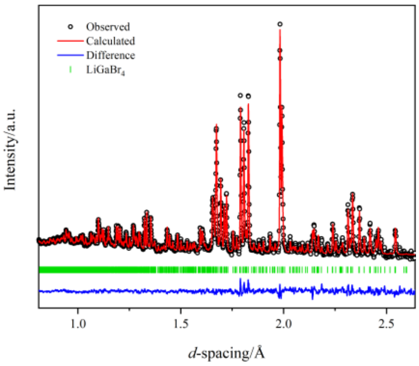

Figure S4. Room temperature profile fit from Rietveld refinement against time-of-flight powder neutron diffraction data for $LiGaBr_4$, collected at Polaris, ISIS. Detector bank 5, ($\langle 2\theta = 146.72°\rangle$), R_{wp} = 2.17 %, R_p = 2.89 %, χ^2 = 2.36.

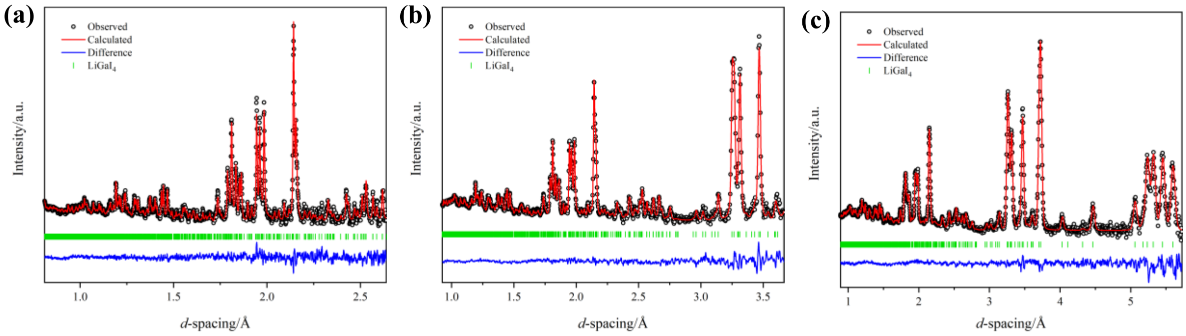

Figure S5. Room temperature profile fits from Rietveld refinement against time-of-flight powder neutron diffraction data for $LiGaI_4$, collected at Polaris, ISIS. **(a)** detector bank 5, ($\langle 2\theta = 146.72°\rangle$), R_{wp} = 1.75 %, R_p = 2.70 %; **(b)** detector bank 4, ($\langle 2\theta = 92.59°\rangle$). R_{wp} = 1.47 %, R_p = 2.33 %, **(c)** detector bank 3, ($\langle 2\theta = 52.51°\rangle$), R_{wp} = 1.85 %, R_p =2.90 %, χ^2 = 1.05.

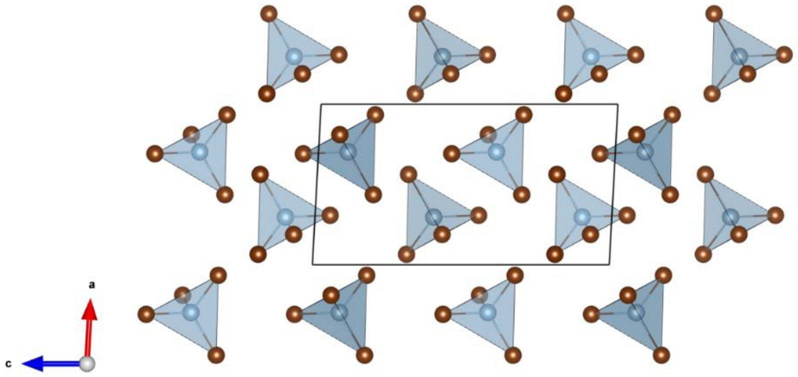

Figure S6. Representation of the isolated $[GaX_4]^-$ tetrahedra projected in the *ac* plane as visualised with VESTA.[1]

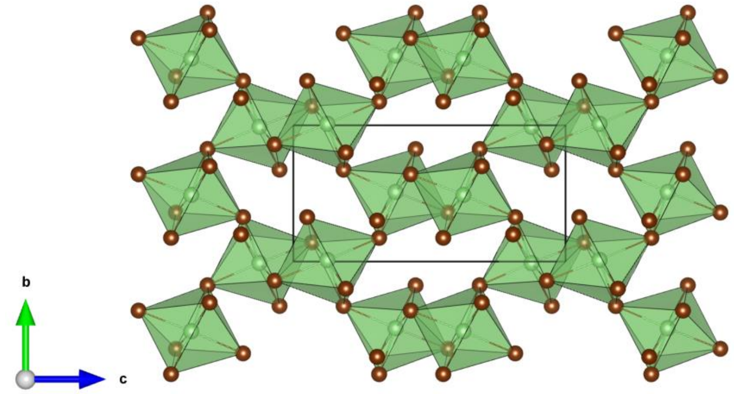

Figure S7. Representation of the linking of the LiX_6 octahedra projected in the *bc* plane as visualised with VESTA.[1]

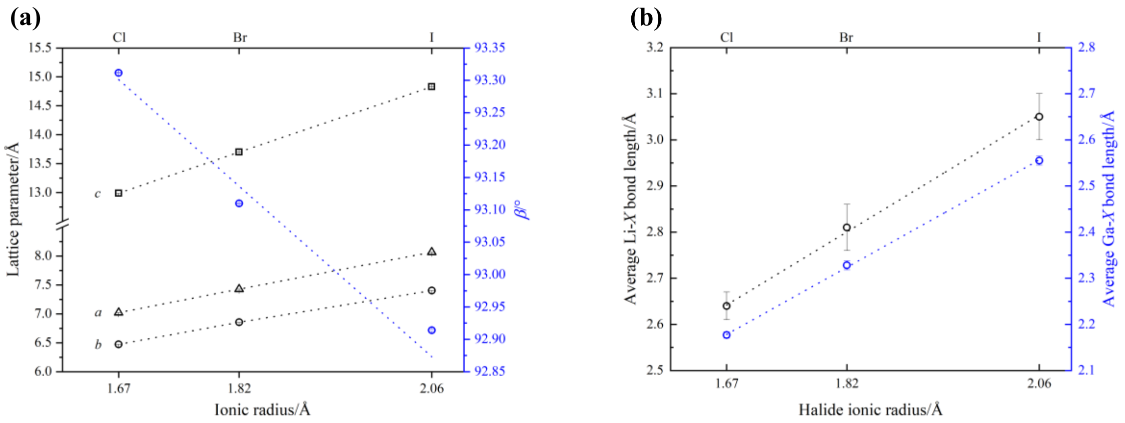

Figure S8. Plots of **(a)** lattice parameters (*a*, *b*, *c* and *β*) and **(b)** mean Li-X and Ga-X bond lengths *vs*. halide ionic radius (with bond lengths calculated from structure parameters refined using PND data).

Table S3. Crystallographic data from Rietveld structure refinement of $LiGaCl_4$ against PND data.

	**Literature[2(a)]**	**PND**
Chemical formula	$LiGaCl_4$	
Crystal system	Monoclinic	
Space group	$P2_1/c$ (14)[(b)]	
Z	4	
a / Å	7.015(5)	7.0211(1)
b / Å	6.467(5)	6.4737(1)
c / Å	12.988(13)	12.9885(2)
β / °	93.36(7)	93.3115(8)
V / Å^3	588.20	589.37(3)
Calculated density, ρ / g cm^{-3}	2.467	2.462
Observations	1706	3821
Parameters	56	68
Phase fraction / %	–	99.010(4)
R_{wp}	0.049	0.0153
R_p	–	0.0256
χ^2	1.171	1.91

[(a)] Single crystal data collected at room temperature.
[(b)] Transformed from setting $P2_1/a$ to $P2_1/c$. TRANS c,-b, a.

Table S4. Atomic parameters of $LiGaCl_4$ obtained from Rietveld structure refinement against PND data.

Atom	Wyckoff	x	y	z	U_{iso} / Å^2 *
Cl1	4e	0.6923(2)	0.3180(4)	0.0495(4)	0.0352*
Cl2	4e	0.8095(2)	0.8648(3)	0.9305(4)	0.03176*
Cl3	4e	0.9272(2)	0.3222(4)	0.8126(4)	0.03578*
Cl4	4e	0.4327(2)	0.1876(4)	0.8103(4)	0.02884*
Ga	4e	0.7023(2)	0.1750(4)	0.8989(3)	0.0230(4)
Li	4e	0.1588(9)	0.508(2)	0.377(2)	0.032(2)

* denotes that U_{iso} values were refined anisotropically.

Table S5. Refined anisotropic displacement parameters of $LiGaCl_4$ from PND data (U_{ij} / Å^2).

Atom	U_{11}	U_{22}	U_{33}	U_{12}	U_{13}	U_{23}
Cl1	0.020(2)	0.043(2)	0.043(2)	0.010(1)	0.003(1)	-0.015(2)
Cl2	0.041(1)	0.020(1)	0.034(1)	-0.001(1)	-0.004(1)	0.002(1)
Cl3	0.033(1)	0.057(2)	0.017(1)	-0.008(2)	0.0057(9)	-0.007(2)
Cl4	0.032(1)	0.035(1)	0.018(1)	-0.007(2)	-0.0092(9)	0.007(1)

Table S6. Selected bond lengths and angles in $LiGaCl_4$ calculated using structure parameters refined from PND data.

Bond	Bond length / Å	Angle	PND / °
**Ga-Cl1**	2.169(3)	**Cl1-Ga-Cl2**	104.6(1)
**Ga-Cl2**	2.176(4)	**Cl1-Ga-Cl3**	109.6(1)
**Ga-Cl3**	2.205(4)	**Cl1-Ga-Cl4**	113.1(1)
**Ga-Cl4**	2.159(3)	**Cl2-Ga-Cl3**	104.1(1)
**Li-Cl1**	2.43(1)	**Cl2-Ga-Cl4**	114.7(2)
**Li-Cl2**	2.66(1)	**Cl3-Ga-Cl4**	110.1(1)
**Li-Cl2**	2.71(1)		
**Li-Cl3**	2.73(1)		
**Li-Cl3**	2.79(1)		
**Li-Cl4**	2.50(1)		

Table S7. Bond-valence sums for $LiGaCl_4$ obtained from Rietveld structure refinement against PND data (GII = 0.040)[3, 4]

Atom	BV Sum
Cl1	-0.99
Cl2	-1.05
Cl3	-0.95
Cl4	-0.98
Ga	3.03
Li	0.94

Table S8. Crystallographic data from Rietveld structure refinement of $LiGaBr_4$ against PND data.

	**Literature[5(a)]**	**PND**
Chemical formula	$LiGaBr_4$	
Crystal system	Monoclinic	
Space group	$P2_1/c$ (14)	
Z	4	
a / Å	7.416(7)	7.4270(2)
b / Å	6.854(7)	6.8548(2)
c / Å	13.696	13.6996(4)
β / °	93.14(8)	93.110(1)
V / Å^3	695.11	696.42(5)
Calculated density, ρ / g cm^{-3}	3.787	3.780
Observations	1414	3806
Parameters	57	48
R_{wp}	0.052	0.0236
R_p	–	0.0303
χ^2	1.35	2.36

[(a)] Single crystal data collected at room temperature.
[(b)] Transformed from setting $P2_1/a$ to $P2_1/c$. TRANS c,-b, a.

Table S9. Atomic parameters of $LiGaBr_4$ obtained from Rietveld structure refinement against PND data.

**Atom**	**Wyckoff**	***x***	***y***	***z***	**U_{iso} / Å^2**
Br1	4*e*	0.6917(3)	0.3257(6)	0.0508(5)	0.0284(9)
Br2	4*e*	0.8108(3)	0.8599(5)	0.9314(6)	0.025(1)
Br3	4*e*	0.9344(3)	0.3263(6)	0.8152(6)	0.034(1)
Br4	4*e*	0.4370(3)	0.1991(5)	0.8075(6)	0.0246(9)
Ga	4*e*	0.7029(2)	0.1759(5)	0.9003(4)	0.0205(6)
Li	4*e*	0.147(2)	0.511(3)	0.375(3)	0.053(4)

Table S10. Selected bond lengths and angles in $LiGaBr_4$ calculated using structure parameters refined from PND data.

Bond	Bond length / Å	Angle	PND / °
**Ga-Br1**	2.309(5)	**Br1-Ga-Br2**	106.1(2)
**Ga-Br2**	2.340(5)	**Br1-Ga-Br3**	108.0(2)
**Ga-Br3**	2.366(5)	**Br1-Ga-Br4**	113.1(2)
**Ga-Br4**	2.295(5)	**Br2-Ga-Br3**	104.0(2)
**Li-Br1**	2.64(2)	**Br2-Ga-Br4**	116.2(2)
**Li-Br2**	2.85(2)	**Br3-Ga-Br4**	108.9(2)
**Li-Br2**	2.80(2)		
**Li-Br3**	2.87(2)		
**Li-Br3**	2.90(2)		
**Li-Br4**	2.79(2)		

Table S11. Bond-valence sums for $LiGaBr_4$ obtained from Rietveld structure refinement against PND data (GII = 0.039)[3, 4]

Atom	BV Sum
Br1	-1.00
Br2	-1.07
Br3	-0.98
Br4	-0.98
Ga	3.05
Li	0.97

Table S12. Crystallographic data from Rietveld structure refinement of $LiGaI_4$ against PND data.

	**Literature[2(a)]**	**PND**
Chemical formula	$LiGaI_4$	
Crystal system	Monoclinic	
Space group	$P2_1/c$ (14)[(b)]	
Z	4	
a / Å	8.081(3)	8.0664(3)
b / Å	7.415(1)	7.4035(3)
c / Å	14.863(5)	14.8298(7)
β / °	92.91(2)	92.914(2)
V / Å^3	889.45	
Calculated density, ρ / g cm^{-3}	4.363	4.388
Observations	1507	5993
Parameters	56	64
R_{wp}	0.038	0.0169
R_p	–	0.0266
χ^2	2.221	1.05

(a) Single crystal data collected at room temperature.
(b) Transformed from setting $P2_1/a$ to $P2_1/c$. TRANS c,-b, a.

Table S13. Atomic parameters of $LiGaI_4$ obtained from Rietveld structure refinement against PND data.

**Atom**	**Wyckoff**	***x***	***y***	***z***	U_{iso} **/ Å^2**
I1	4*e*	0.6879(3)	0.3266(8)	0.0545(6)	0.028(1)
I2	4*e*	0.8121(3)	0.8516(6)	0.9306(5)	0.022(1)
I3	4*e*	0.9356(3)	0.3322(7)	0.8162(6)	0.032(1)
I4	4*e*	0.4361(3)	0.1989(6)	0.8076(5)	0.018(1)
Ga	4*e*	0.7047(2)	0.1740(5)	0.9016(3)	0.0206(6)
Li	4*e*	0.143(1)	0.505(2)	0.380(2)	0.062(4)

Table S14. Selected bond lengths and angles in $LiGaBr_4$ calculated using structure parameters refined from PND data.

Bond	Bond length / Å	Angle	PND / °
**Ga-I1**	2.544(5)	**I1-Ga-I2**	107.2(2)
**Ga-I2**	2.568(5)	**I1-Ga-I3**	107.9(2)
**Ga-I3**	2.586(5)	**I1-Ga-I4**	112.2(2)
**Ga-I4**	2.523(5)	**I2-Ga-I3**	104.9(2)
**Li-I1**	2.89(2)	**I2-Ga-I4**	115.7(2)
**Li-I2**	3.03(2)	**I3-Ga-I4**	108.4(2)
**Li-I2**	3.00(2)		
**Li-I3**	3.18(2)		
**Li-I3**	3.13(2)		
**Li-I4**	3.05(2)		

Table S15. Bond-valence sums for $LiGaI_4$ obtained from Rietveld structure refinement against PND data (GII = 0.046)[3, 4]

Atom	BV Sum
I1	-0.99
I2	-1.09
I3	-0.98
I4	-0.97
Ga	3.05
Li	0.99

Table S16. Comparative summary of crystallographic parameters for $LiMX_4$ materials (M = Al, Ga; X = Cl, Br, I).[6]

$LiMX_4$	Average M-X bond distance / Å	MX_4 polyhedral volume / Å$^{3(a)}$	Average Li-X bond distance /Å	LiX_6 polyhedral volume / Å$^{3(a)}$
$LiAlCl_4$	2.144(8)	5.034	2.65(1)	24.169
$LiGaCl_4$	2.177(4)	5.262	2.64(1)	24.011
$LiAlBr_4$	2.352(8)	6.624	2.83(1)	29.839
$LiGaBr_4$	2.328(3)	6.427	2.81(1)	29.090
$LiAlI_4$	2.560(7)	8.703	3.08(1)	38.480
$LiGaI_4$	2.555(3)	8.517	3.05(1)	37.372

[(a)] Calculated with VESTA.[1]

Table S16. (*continuation*)

$LiMX_4$	*a* / Å	*b* / Å	*c* / Å	β / °	V / Å^3
$LiAlCl_4$	7.0011(3)	6.5042(3)	12.9884(6)	93.321(2)	590.45(6)
$LiGaCl_4$	7.0211(1)	6.4737(1)	12.9885(2)	93.3115(8)	589.37(3)
$LiAlBr_4$	7.4358(4)	6.8930(4)	13.7385(9)	93.212(3)	703.1(1)
$LiGaBr_4$	7.4270(2)	6.8548(2)	13.6996(4)	93.110(1)	696.42(5)
$LiAlI_4$	8.0771(6)	7.4314(6)	14.882(1)	93.070(4)	892.0 (1)
$LiGaI_4$	8.0664(3)	7.4035(3)	14.8298(7)	92.914(2)	884.5(1)

Thermal stabilities

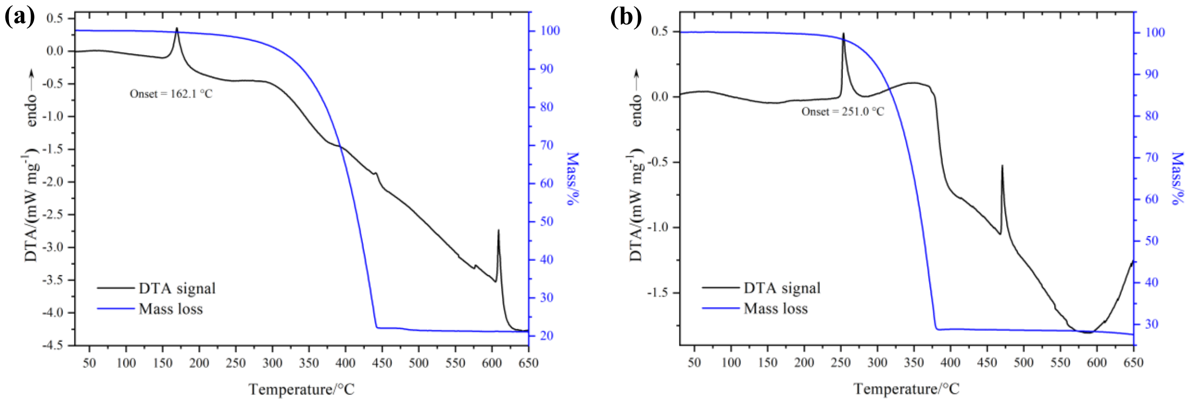

Figure S9. TG-DTA profiles of **(a)** $LiGaCl_4$ and **(c)** $LiGaI_4$ heated to 650 °C at 5 °C min^{-1} under flowing Ar (60 mL min^{-1}). Under these conditions, $LiGaCl_4$ melts at 162.1 °C followed by decomposition at *ca.* 308 °C, and $LiGaI_4$ melts at 251. °C followed by immediate decomposition.

Table S17. Thermal properties of $LiGaX_4$ (X = Cl, Br, I).

Material	Melting point/°C	Literature/°C	Decomposition temperature/°C
$LiGaCl_4$	162.1	167[2]	~ 308
$LiGaBr_4$	203.6	205[5]	~ 277
$LiGaI_4$	251.0	239[2]	–

Table S18. Evaluation of proposed decomposition mechanism of $LiGaX_4$ (X = Cl, Br, I) from STA data.

Decomposition reaction	Weight loss / %		Li*X* melting point/°C	
$LiGaX_4 \rightarrow LiX + GaX_3$	Theory	Experiment	Literature[7]	Experiment
X = Cl	78.08	77.49	610	606.7
X = Br	80.60	79.02	550	550.4
X = I	77.09	71.51	469	469.1

Electronic structure

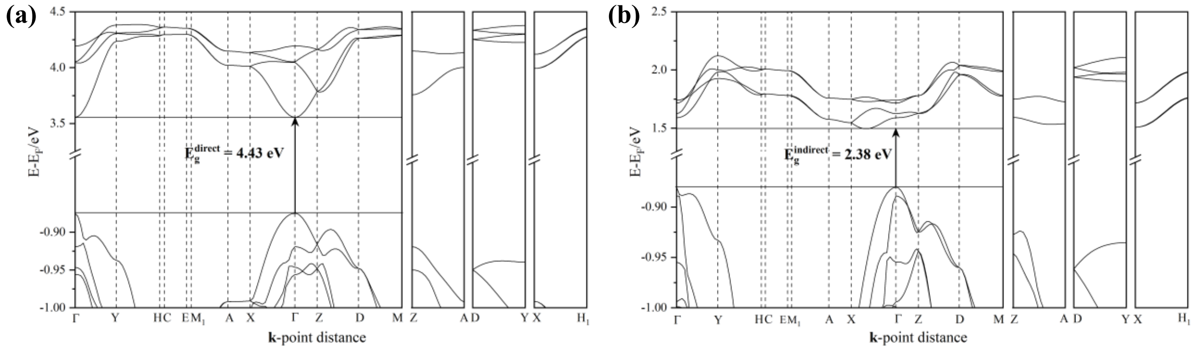

Figure S10. Computed (DFT/PBE) band structures of **(a)** $LiGaCl_4$ and **(b)** $LiGaI_4$, with band energies scaled to the Fermi level (E_F). High symmetry **k**-points are noted as well as the indirect gap region between VBM and CBM.

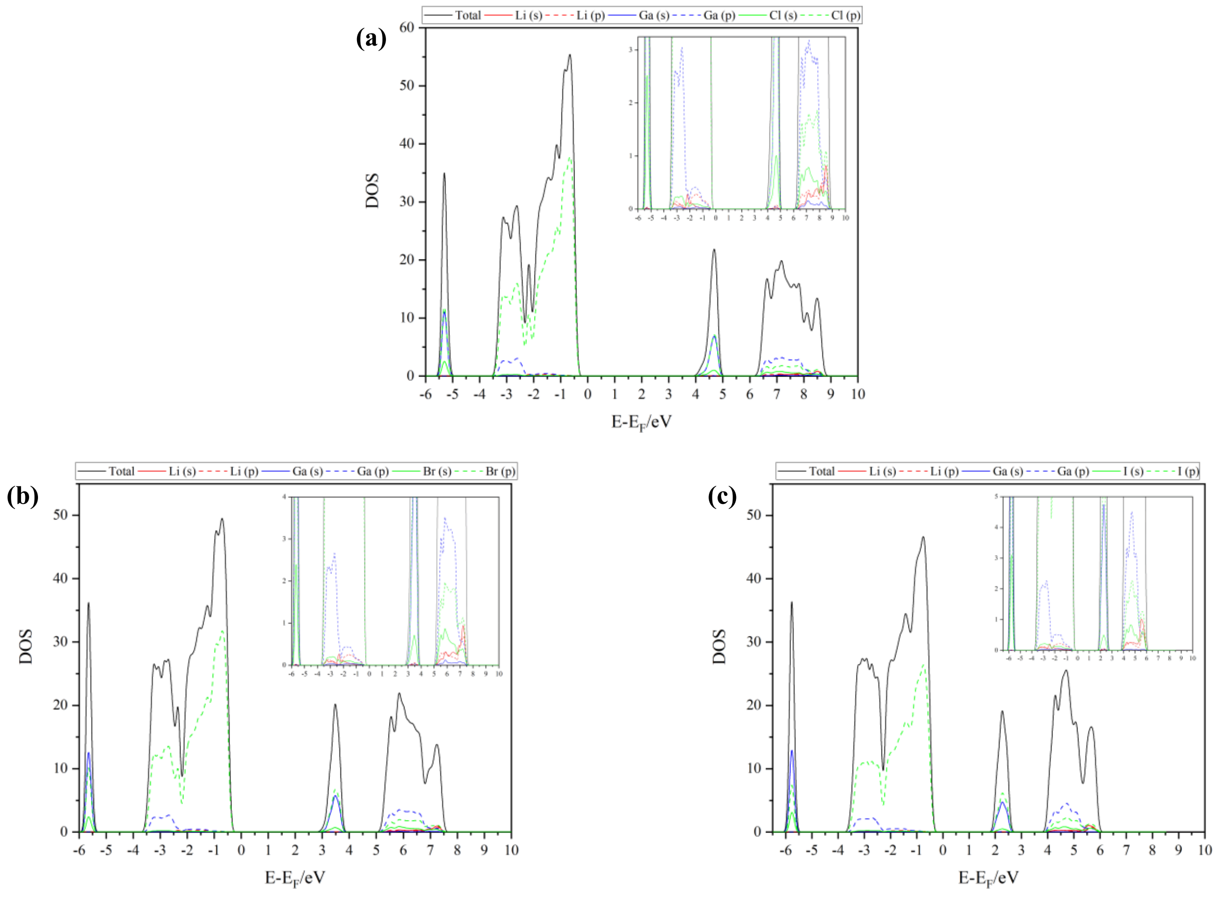

Figure S11. Computed (DFT/PBE) total and atom-projected electronic density of states (DOS) of **(a)** $LiGaCl_4$, **(b)** $LiGaBr_4$ and **(c)** $LiGaI_4$.

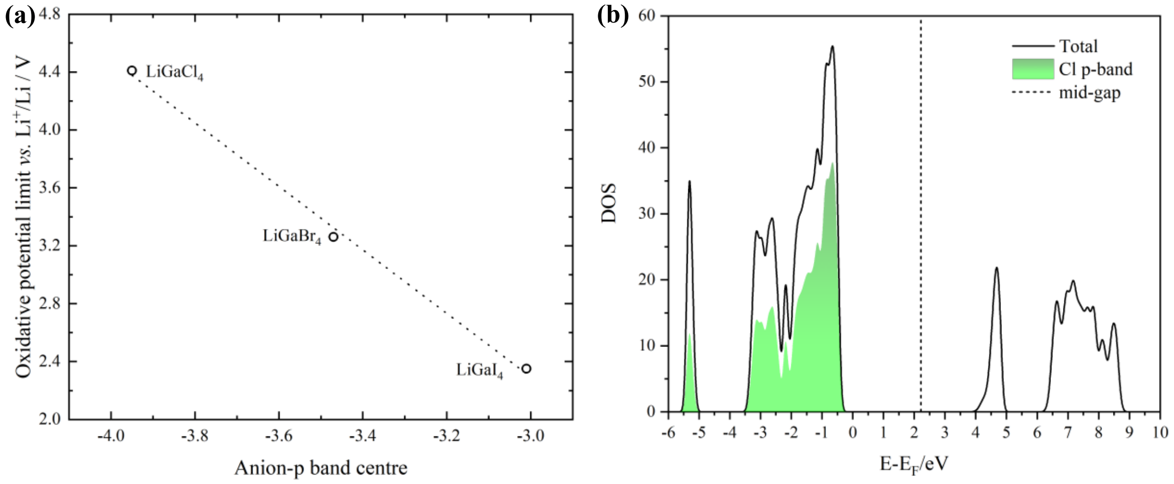

Figure S12. (a) Correlation between computed oxidative potential limit[8] and computed (DFT/PBE) anion p-band centre with respect to the mid-gap in $LiGaX_4$ (X = Cl, Br, I) materials. The dotted line corresponds to a linear fit (R^2 = 0.997). **(b)** Example of Cl-projected electronic DOS in $LiGaCl_4$. The Cl p-band centre was computed by integrating over the occupied states closer to the VBM with respect to the mid-gap energy indicated by a dashed line.

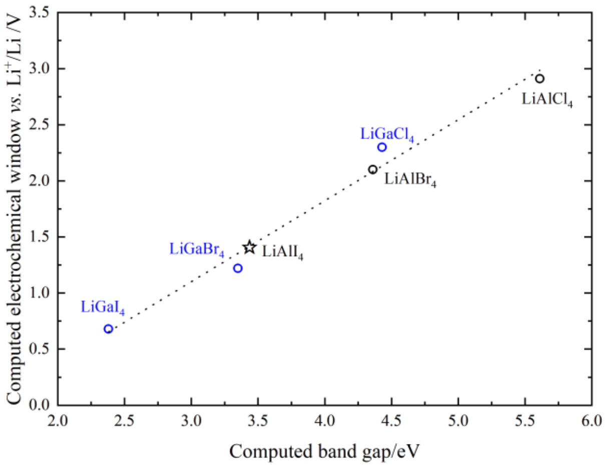

Figure S13. Correlation between computed EW values available in the literature[8] and computed band gaps in $LiMX_4$ (M = Al, Ga; X = Cl, Br, I) materials. The EW for $LiAlI_4$ is predicted to be 1.4 V *vs.* Li^+/Li. The dotted line corresponds to a linear fit (R^2 = 0.983).

Raman spectra

Table S19. Experimental and computed Raman (DFT/FD/PBE) frequencies of $LiGaX_4$ (X = Cl, Br I). λ_{laser} = 1064 nm.

$LiGaCl_4$			$LiGaBr_4$			$LiGaI_4$		
Experiment ω/cm^{-1}	FD/PBE ω/cm^{-1}	Mode	Experiment ω/cm^{-1}	FD/PBE ω/cm^{-1}	Mode	Experiment ω/cm^{-1}	FD/PBE ω/cm^{-1}	Mode
	395.2	B_g		306.0	B_g		255.4	B_g
396.8	390.1	A_g		302.6	A_g		255.3	A_g
	372.7	B_g		285.6	B_g		239.9	B_g
381.3	371.2	A_g		284.6	A_g		235.3	A_g
	345.9	A_g		261.1	A_g		215.1	A_g
	344.0	B_g		255.5	B_g		209.9	B_g
348.6	331.5	A_g		223.9	B_g		190.1	A_g
	330.2	B_g		223.2	A_g		189.9	B_g
	266.0	B_g		205.3	B_g		175.4	B_g
	265.4	A_g		204.3	A_g		173.8	A_g
	247.6	B_g		197.9	B_g		147.2	B_g
	245.1	A_g	209.7	197.1	A_g	146.1	138.0	A_g
	203.6	B_g		165.2	B_g		137.5	B_g
	183.4	A_g		134.9	A_g		124.6	A_g
167.3	168.8	A_g		113.7	B_g		85.3	B_g
	167.1	B_g	111.4	111.4	A_g	80.5	82.1	A_g
	161.4	B_g		108.9	B_g		79.0	B_g
148.0	147.0	B_g		101.8	A_g		76.6	A_g
	143.5	A_g		95.6	B_g		69.9	B_g
	134.7	A_g	97.9	90.2	A_g	72.8	67.9	A_g
	125.2	B_g	80.5	81.2	A_g		60.9	A_g
121.0	119.8	B_g		80.5	B_g		58.1	B_g
	117.2	A_g		77.1	B_g		57.5	B_g
	114.5	A_g		74.8	A_g		55.1	A_g
	95.6	B_g		65.7	B_g		49.8	B_g
	90.9	A_g		63.8	A_g		48.3	A_g
	80.5	B_g		57.5	B_g		45.1	B_g
	78.1	A_g		54.2	A_g		41.6	A_g
	66.8	A_g		49.2	A_g		38.9	A_g
	65.5	B_g		48.6	B_g		38.8	B_g
	63.6	A_g		46.5	B_g		36.6	B_g
	59.0	B_g		45.9	A_g		34.8	A_g
	53.6	B_g		39.1	B_g		31.0	B_g
	48.6	B_g		35.7	A_g		29.1	A_g
	47.8	A_g		33.7	B_g		26.4	B_g
	32.6	A_g		23.5	A_g		19.3	A_g

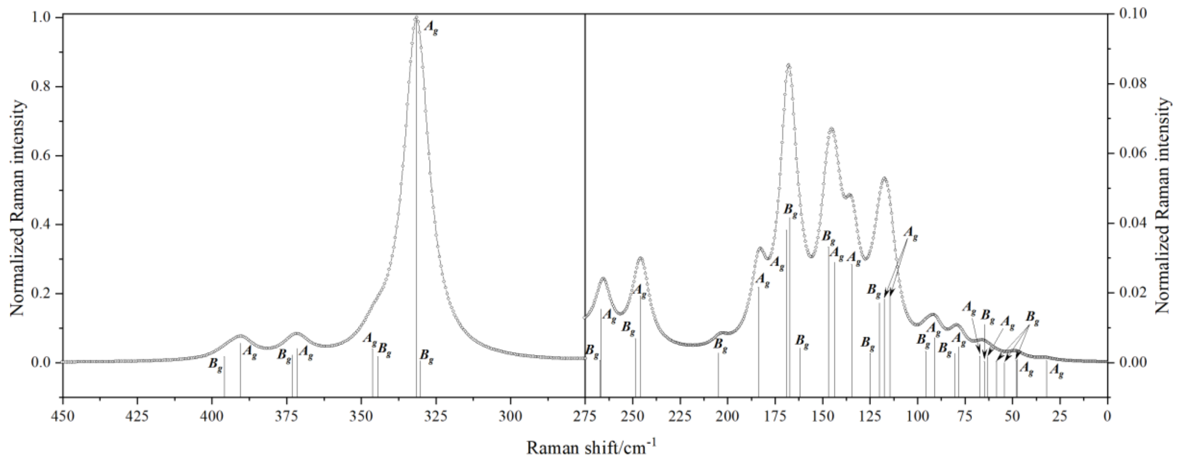

Figure S14. Theoretically simulated (DFT/FD/PBE) Raman spectrum of $LiGaCl_4$ showing the underlying 36 Raman active modes

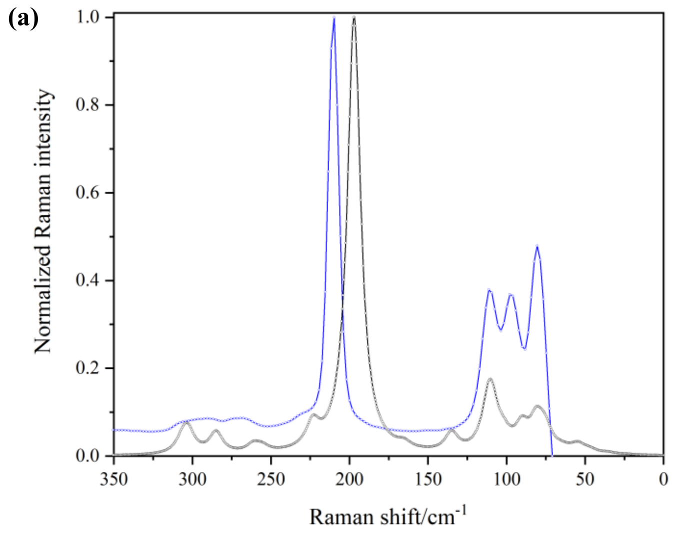

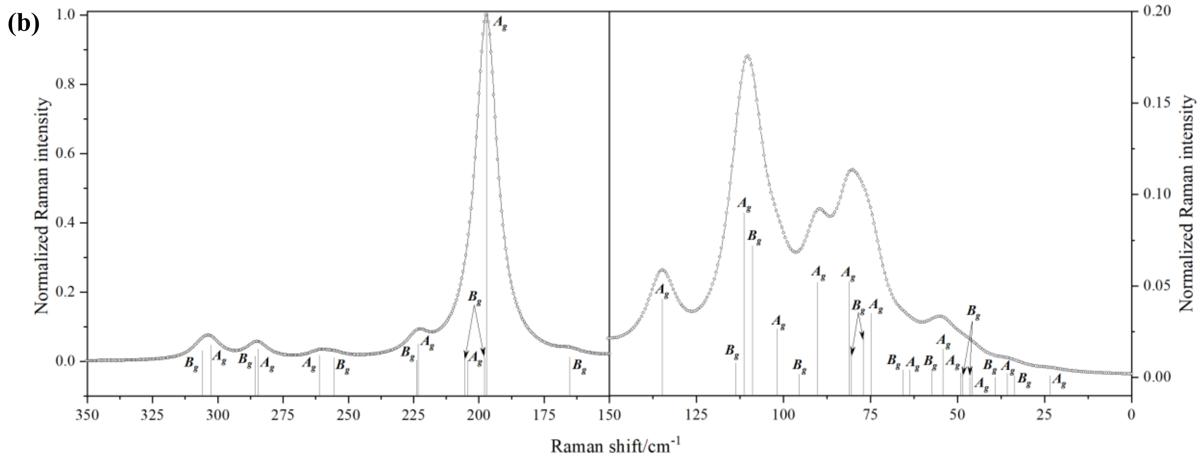

Figure S15. **(a)** Theoretically simulated (DFT/FD/PBE; black line) *vs.* experimentally observed (blue line) Raman spectrum of $LiGaBr_4$ (λ_{laser} = 1064 nm), showing **(b)** the underlying 36 Raman active modes.

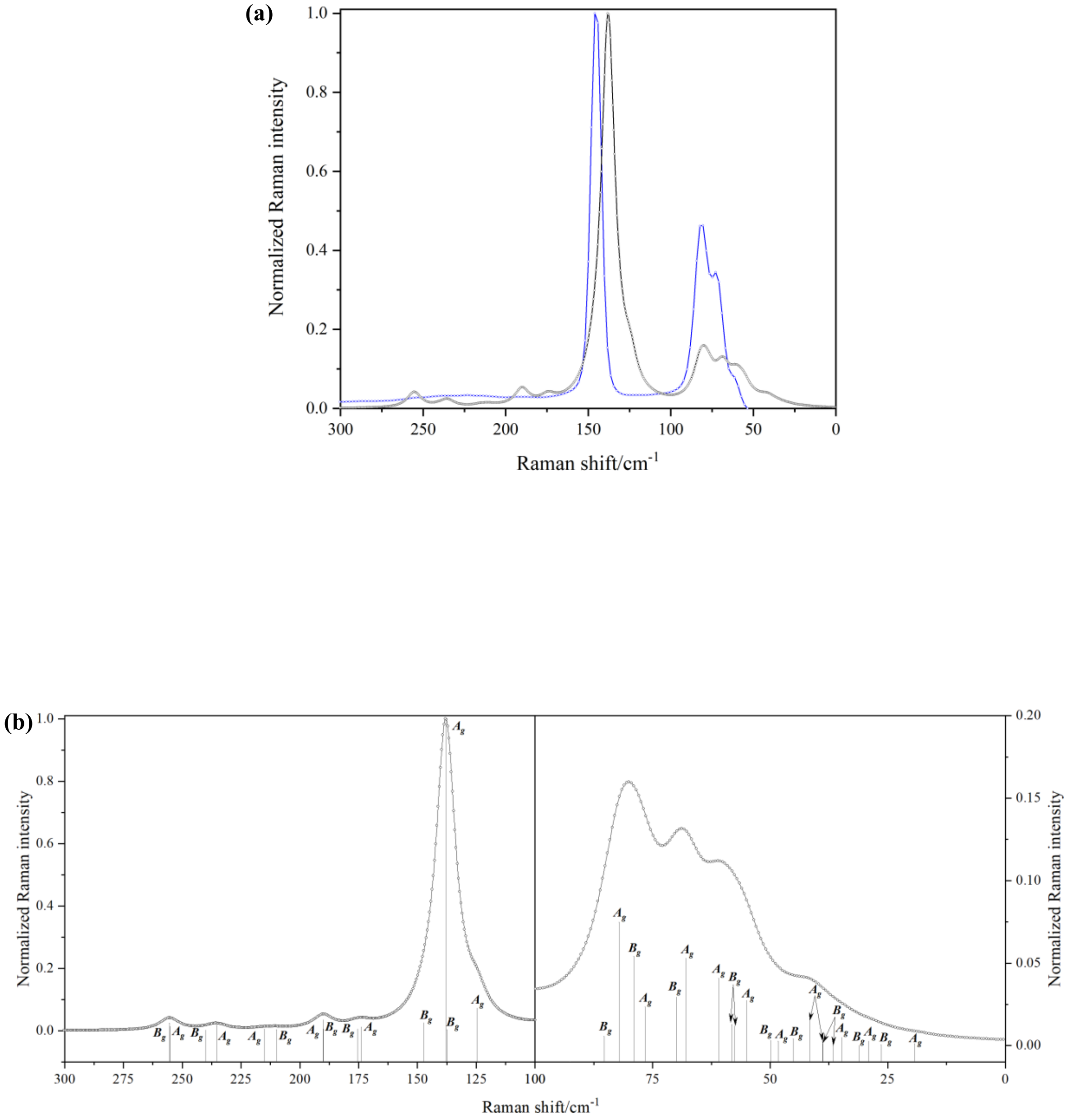

Figure S16. (a) Theoretically simulated (DFT/FD/PBE; black line) *vs.* experimentally observed (blue line) Raman spectrum of $LiGaI_4$ (λ_{laser} = 1064 nm), showing **(b)** the underlying 36 Raman active modes.

N.B.: The observed mismatch between the simulated and experimental Raman spectra for $LiGaBr_4$ and $LiGaI_4$ likely arises from a combination of vibrational anharmonicity, bonding polarisability, and microstructural effects in the experimental samples. In contrast, the lighter and more rigid $LiGaCl_4$ lattice yields better agreement under the harmonic approximation. In addition, it should be noted that GGA-based functionals are generally less accurate for heavy elements, where semicore electrons are more contracted and thus not well described by these approaches. This limitation may also contribute to the poorer agreement observed for Br and I.

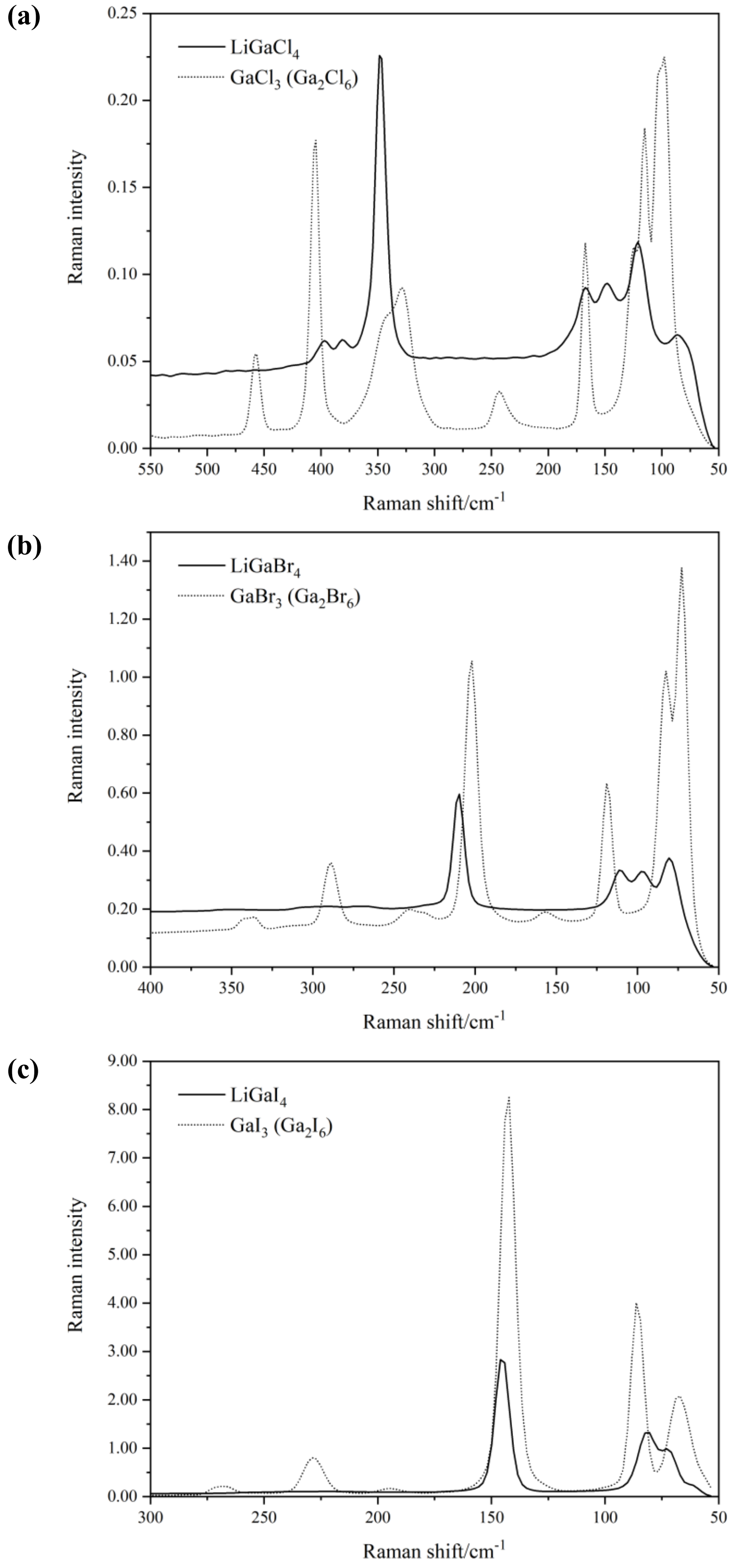

Figure S17. Comparison of the experimental room temperature Raman spectra of $LiGaX_4$ *vs.* GaX_3 starting materials. **(a)** X = Cl, **(b)** X = Br, and **(c)** X = I. λ_{laser} = 1064 nm.

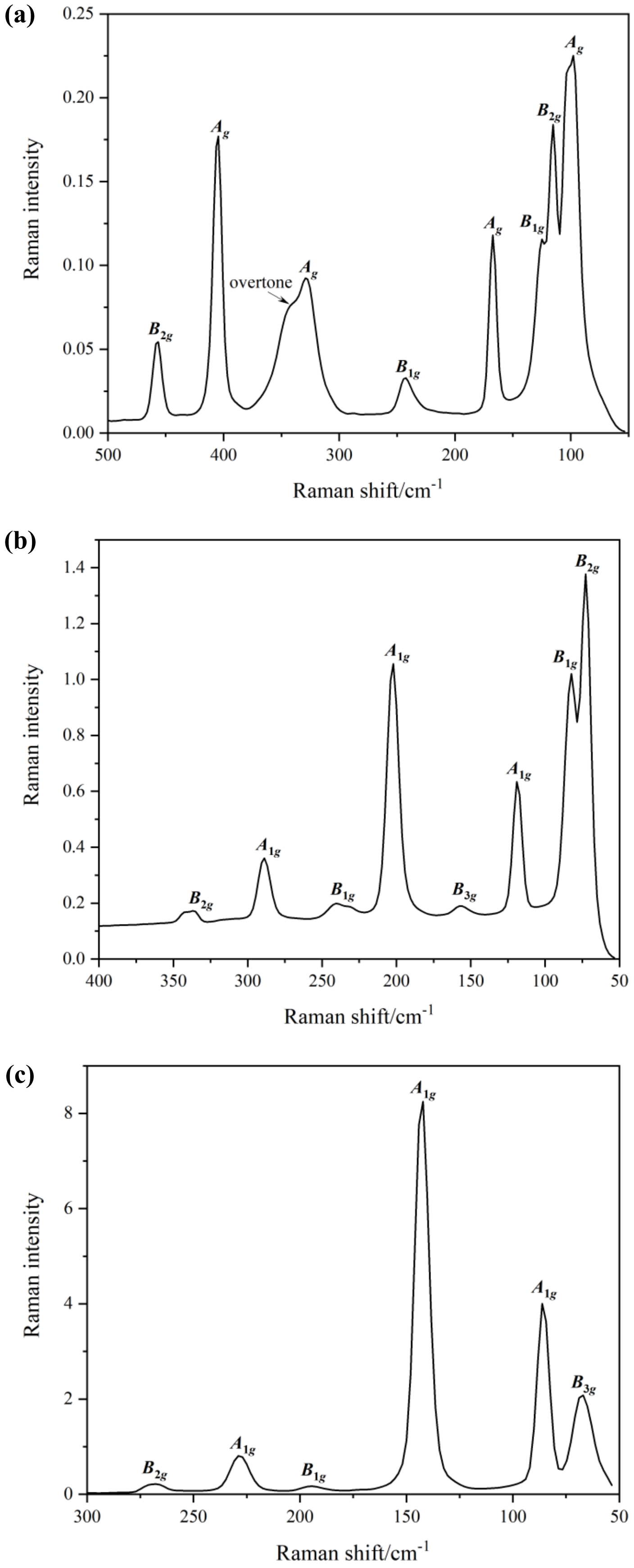

Figure S18. Room temperature Raman spectra of **(a)** $GaCl_3$, **(b)** $GaBr_3$ and **(c)** GaI_3, showing the assignment of their wave numbers (λ_{laser} = 1064 nm).

Table S20. Assignment of the Raman wave numbers (in cm^{-1}) for GaX_3 (X = Cl, Br, I)[1,2]

**$GaCl_3(Ga_2Cl_6)$**	**Assignment**	**$GaBr_3(Ga_2Br_6)$**	**Assignment**	**$GalI_3(Ga_2I_6)$**	**Assignment**
457	B_{2g}	337	B_{2g}	268	B_{2g}
405	A_g	289	A_{1g}	229	A_{1g}
342	*overtone*	241	B_{1g}	194	B_{1g}
329	A_g	202	A_{1g}	142	A_{1g}
167	A_g	158	B_{3g}	86	A_{1g}
125	B_{1g}	119	A_{1g}	67	B_{3g}
115	B_{2g}	82	B_{1g}		
98	A_g	73	B_{2g}		

Macroscopic ion transport

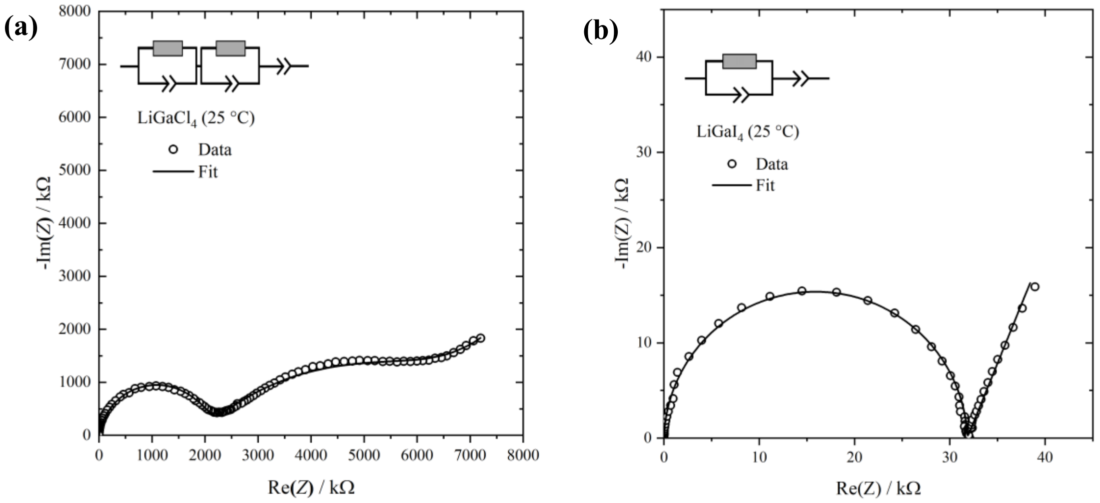

Figure S19. Room temperature Nyquist plots of **(a)** $LiGaCl_4$ and **(b)** $LiGaI_4$, showing the impedance responses (open circles) and fits (solid lines). The relative densities of the pellets are 79% and 90 %, respectively.

Table S21. Selected transport data for $LiGaX_4$ (X = Cl, Br, I) materials as comparted to experimental values from the literature.

	$\sigma_{RT} \times 10^6$ / S cm^{-1}		E_a / eV	
Material	This work	Literature	This work	Literature
$LiGaCl_4$	0.157(2)	–	1.05(3)[(b)], 0.479(33)[(c)]	–
$LiGaBr_4$	4.87(4)	7[9(a)], 20[10(b)]	0.49(3)[(b)], 0.50,[10(b)] 0.352(64)[(c)]	0.37[9(d)]
$LiGaI_4$	0.633(4)	–	0.63(3)[(b)], 0.268(23)[(c)]	–

[(a)] EIS measurement (annealed sample).
[(b)] EIS measurement (ball milled sample).
[(c)] μ^+SR measurement (ball milled sample).
[(d)] ^{7}Li NMR measurements (annealed sample).

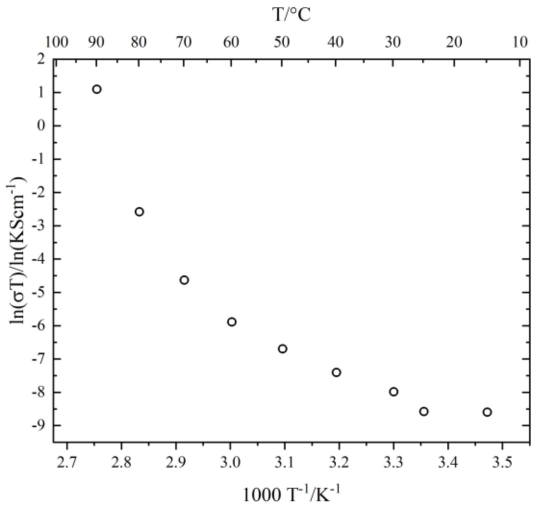

Figure S20. Arrhenius plot of conductivity values obtained from temperature-dependent impedance spectroscopy between 15-90 °C for $LiGaI_4$.

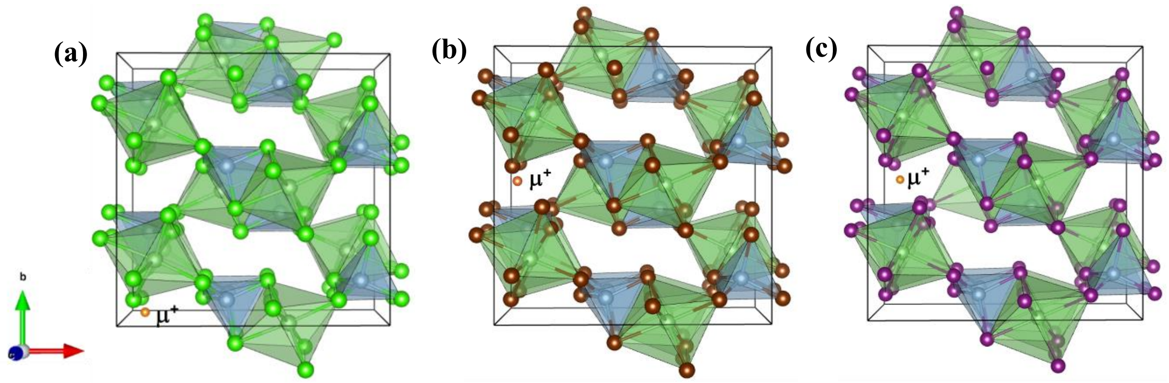

Figure S21. Calculated muon stopping sites for **(a)** $LiGaCl_4$, **(b)** $LiGaBr_4$ and **(c)** $LiGaI_4$. Li (dark green spheres), Cl (green spheres), Br (brown spheres), I (purple spheres), μ^+ (orange spheres).

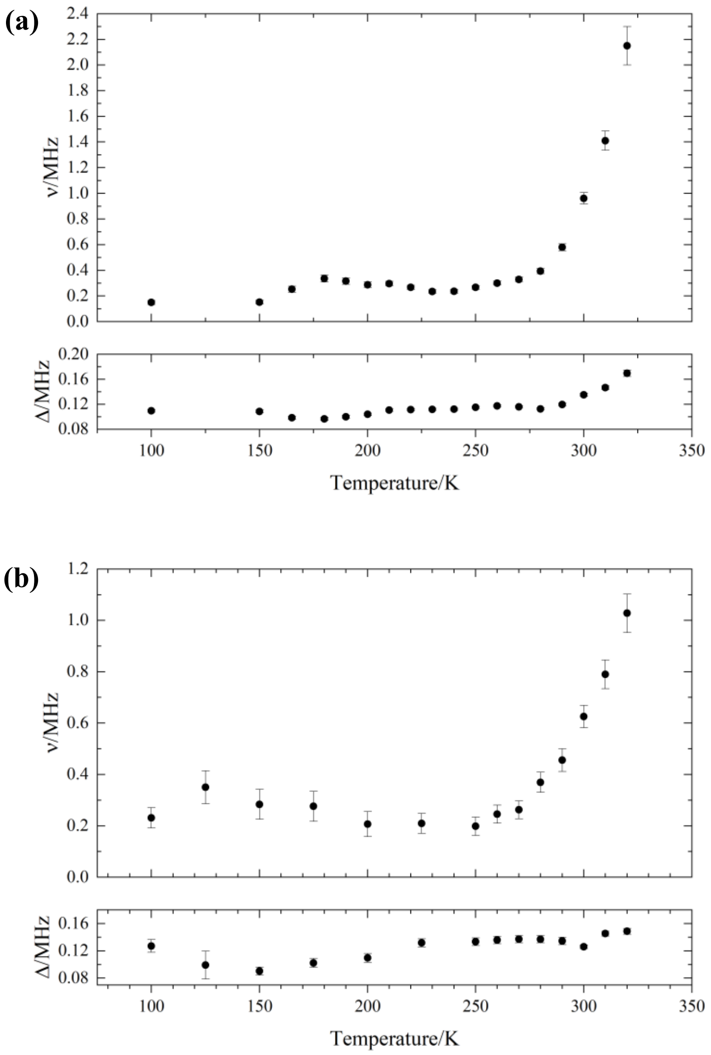

Figure S22. Temperature dependences of the (top) fluctuation rate, ν, and (bottom) static widths of the local field distribution, Δ, using Equation 2, for **(a)** $LiGaCl_4$ and **(b)** $LiGaI_4$.

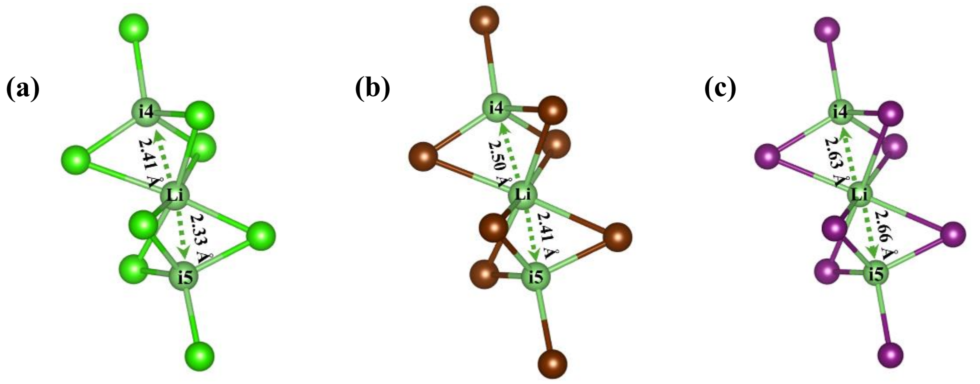

Figure S23. Jump distances and coordination of the interstitial sites around the Li lattice site in **(a)** $LiGaCl_4$, **(b)** $LiGaBr_4$ and **(c)** $LiGaI_4$. Li and i4 (dark green spheres), Cl (green spheres), Br (brown spheres) and I (purple spheres).

Conduction mechanisms

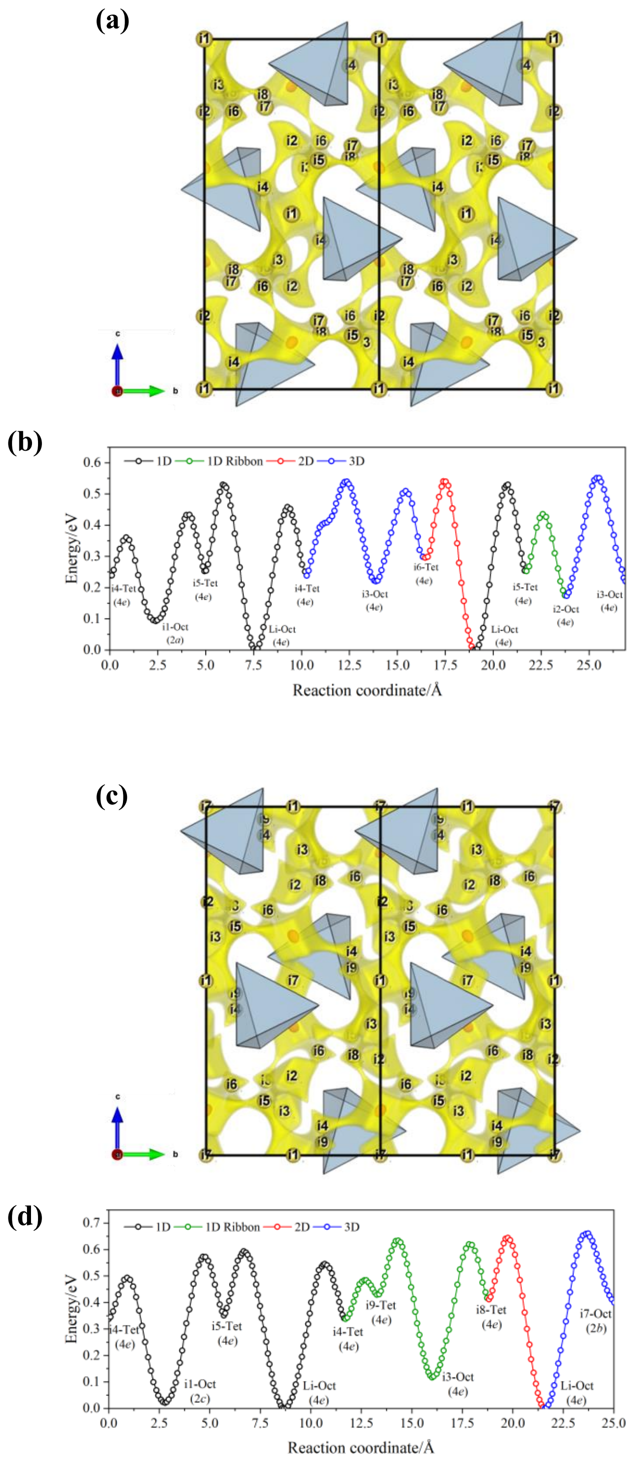

Figure S24. (a, c) BVSE map showing Li migration pathways in a (100) projection of the $LiGaX_4$ (X = Cl, and I) structures, as visualised with VESTA[1]. The highest isosurface levels of 0.56 eV (X = Cl) and 0.67 eV (X = I) over the global minimum are shown in yellow. Red dots indicate octahedral Li lattice sites and yellow spheres indicate tetrahedral/octahedral interstitial sites. **(b, d)** BVSE models of migration barriers for $LiGaX_4$ derived reference data (ICSD. $LiGaCl_4$, collection code 60849; and $LiGaI_4$, collection code 60850). The relative site energy is zero for Li lattice sites.

References

1. K. Momma and F. Izumi, *J. Appl. Crystallogr.*, 2011, **44**, 1272–1276.
2. W. Hönle, B. Hettich and A. Simon, *Z. Naturforsch., B*, 1987, **42**, 248.
3. A. Salinas-Sanchez, J. L. Garcia-Muñoz, J. Rodriguez-Carvajal, R. Saez-Puche and J. L. Martinez, *J. Solid State Chem.*, 1992, **100**, 201–211.
4. H. Chen, L. Wong and S. Adams, *Acta Crystallogr., Sect. B*, 2019, **75**, 18–33.
5. W. Hönle and A. Simon, *Z. Naturforsch., B*, 1986, **41**, 1391.
6. N. Flores-González, N. Minafra, G. Dewald, H. Reardon, R. I. Smith, S. Adams, W. G. Zeier and D. H. Gregory, *ACS Mater. Lett.*, 2021, **3**, 652–657.
7. W. M. Haynes, *CRC Handbook of Chemistry and Physics*, 97th edn, CRC Press, 2016.
8. S. Wang, Q. Bai, A. M. Nolan, Y. Liu, S. Gong, Q. Sun and Y. Mo, *Angew. Chem. Int. Ed.*, 2019, **58**, 8039–8043.
9. Y. Tomita, H. Ohki, K. Yamada and T. Okuda, *Solid State Ionics*, 2000, **136–137**, 351–355.
10. L. Gao, F. Zhong, Y. Tong, S. Zhang, J. You, H. Wei, X. Yu, S. Xu and G. Zhao, *ACS Appl. Energy Mater.*, 2022, **5**, 10604–10610.
